# THESES-M: protocol for the development of a reporting guideline for medical theses

**DOI:** 10.1371/journal.pone.0354899

**Published:** 2026-08-07

**Authors:** Carlos Rivero-López, Ana Elena Limón-Rojas, Ricardo Octavio Morales-Carmona, Claudia Jimena Vilchis-Macedo, Liz Hamui-Sutton, Magali Fabiola Vega-Rodríguez, Luisa Fernanda Romero-Henríquez, Geovani López-Ortiz

**Affiliations:** 1 Division of Postgraduate Studies, Faculty of Medicine, National Autonomous University of Mexico, Mexico City, Mexico; 2 Department of Family Medicine, Faculty of Medicine, National Autonomous University of Mexico, Mexico City, Mexico; 3 Center for Teacher Professional Development in Mexico City, Ministry of Public Education, Mexico City, Mexico; Laval University, CANADA

## Abstract

**Introduction:**

Medical theses represent one of the most widely produced yet least standardized forms of health sciences research. Their structure and reporting vary substantially across institutions, programs, and regions, and no formal reporting criteria exist to guide their preparation. This variability affects the consistency and evaluation of medical theses and may influence subsequent research reporting practices. This study describes the protocol for the development of THESES-M (Transparent and Holistic Evaluation Standards for Educational Studies in Medicine), a reporting guideline for medical theses.

**Materials and methods:**

THESES-M will be developed in accordance with the guidance proposed by Moher et al. and the recommendations of the EQUATOR Network. The study will include a literature review to identify structural components of medical theses, generation of a preliminary list of reporting items, and three Delphi rounds with an expert panel using a 1–10 Likert scale. Consensus thresholds are predefined (≥80% rating ≥7 for inclusion; ≥ 80% rating ≤4 for exclusion). Agreement and stability will be assessed using median scores, interquartile ranges, and Kendall’s coefficient of concordance. Items without consensus will be discussed in an in-person consensus meeting. The study outputs will include a consensus-based checklist and an explanation and elaboration document. Both will undergo pilot testing with medical students to assess clarity, feasibility, and usability.

**Ethics and dissemination:**

Ethical approval was obtained from the Research and Ethics Committees of the Faculty of Medicine, National Autonomous University of Mexico (FM/DI/068/2025). Study findings will be disseminated through peer-reviewed publications and submission of the final guideline to the EQUATOR Network. THESES-M is registered with the EQUATOR Network as a reporting guideline under development.

## Introduction

Medical theses are formal research documents produced as part of training in the health sciences. Although they are not mandatory in all medical education systems, in several settings, including countries in Europe, Latin America, Asia, and Africa, they remain part of training at both undergraduate and postgraduate levels, including residency. Where required, they are a prerequisite for graduation [1–5]. Their preparation involves the integration of methodological, argumentative, and scientific writing skills, and they often represent one of the earliest structured forms through which research is documented and communicated [1,2,6,7]. Despite their use in these contexts, medical theses are reported inconsistently across institutions, programs, and regions [3,8,9]. This heterogeneity affects the clarity, structure, and evaluability of these documents and may influence how research is communicated in later academic and professional settings.

The absence of standardized reporting criteria coexists with longstanding concerns regarding the quality, use, and dissemination of theses [8,9]. Without explicit guidance on what information should be reported and how it should be organized, medical theses may differ substantially in structure, content, and methodological detail, even when addressing the same study design. This variability weakens the consistency of the final document and may contribute to the low proportion of theses that are subsequently converted into scientific publications [5,10,11]. In this context, available evidence, including medical student master’s theses, shows marked heterogeneity in thesis-to-publication rates, ranging from 0.8% to 33% [5,11]. Deficiencies in the methodological reporting of medical theses have been documented across institutional settings, and thesis supervisors have identified structural and methodological problems as primary factors in the failure of thesis projects [8,10]. These findings suggest that the absence of explicit reporting criteria has measurable consequences for the consistency and evaluability of these documents.

In response to this gap, many institutions have developed internal guidance for thesis preparation. However, these documents are usually limited in scope and tend to focus on formal presentation requirements, such as cover-page format, citation style, or length, rather than on minimum standards for methodological reporting. This contrasts with the progress achieved in other areas of medical research, where reporting guidelines have been developed for multiple study designs, mainly through the EQUATOR Network [12]. These initiatives have established minimum standards for transparency, reproducibility, and completeness in scientific reporting, thereby supporting critical appraisal and use in evidence synthesis [13–15]. These principles are also relevant to medical theses, where clear reporting of the study rationale, methods, results, and ethical considerations is essential for adequate academic evaluation and scientific communication [3,9]. Extending these standards to medical theses requires recognition that academic work produced during training also demands explicit and complete reporting.

Medical theses share these reporting needs, but they also include components that extend beyond the description of the study itself, such as the statement of the problem, the study rationale, and the regulatory and ethical considerations that support the work. In this context, the THESES-M initiative (Transparent and Holistic Evaluation Standards for Educational Studies in Medicine) proposes the development of a reporting guideline for medical theses. The initiative aims to establish a structured framework for defining the essential sections and content of these documents across different levels of medical training and methodological approaches.

## Materials and methods

### Scope of the guideline

In this study, medical theses are defined as formally supervised academic research projects developed within undergraduate or postgraduate medical training programs and submitted as a requirement for the award of an academic degree. This definition includes theses addressing different thematic areas (clinical, educational, health systems, or other medicine-related fields), regardless of methodological approach, as part of the research processes embedded in medical education. In this context, undergraduate training refers to entry-level medical education leading to a medical degree, whereas postgraduate training includes residency programs, master’s degrees, and doctoral education in medicine.

### Aim

To develop a reporting guideline for medical theses using a structured process that includes literature review, Delphi consensus rounds, a consensus meeting, and pilot testing.

### Study design

This is a methodological study for the development of a reporting guideline, conducted in accordance with the guidance proposed by Moher et al. and the recommendations of the EQUATOR Network [16]. The process is organized into sequential phases with iterative components, including a literature review, Delphi rounds, a consensus meeting, and pilot testing. This approach is intended to identify, appraise, and refine the items required for the final guideline.

### Setting

The study will be conducted at the Faculty of Medicine of the National Autonomous University of Mexico (UNAM). Consensus activities are expected to take place primarily in person at the institution’s academic facilities.

### EQUATOR registration

The THESES-M guideline is registered in the Reporting Guidelines Under Development section of the EQUATOR Network [17].

### Development framework

THESES-M will be developed in accordance with the five phases described by Moher et al [16]:

Initial steps: identification of need, definition of scope, and preliminary literature review.
Pre-meeting activities: generation of the preliminary list of items for checklist development, selection of experts, distribution of materials, and completion of three Delphi rounds.
Consensus meeting: structured discussion and in-person refinement of the items.
Post-meeting activities: consolidation of agreements and pilot testing.
Knowledge translation and post-publication activities: strategies for dissemination, institutional adoption, and future updating.

### Phase 1: Initial steps

#### Identification of need and scope.

THESES-M is intended to address the absence of reporting standards specifically designed for medical theses. It seeks to define the minimum reporting criteria applicable to degree-granting theses in medicine, regardless of level of training or methodological approach.

#### Literature review.

A literature review using systematic procedures will be undertaken to identify recurring components in the reporting of medical theses. The search will be carried out independently by CRL and GLO in PubMed, ERIC, Google Scholar, and institutional repositories and websites of medical schools, using combinations of English- and Spanish-language terms such as “medical thesis,” “medical theses,” “medical dissertation,” “thesis guidelines,” “dissertation guidelines,” “medical education,” “reporting,” and “structure,” and their Spanish equivalents. Searches will include documents available up to 31 August 2026, with no lower date limit. Institutional repositories and websites will be searched through official university and medical school websites, using the same terms combined with institution names and by reviewing sections related to regulations, degree requirements, postgraduate programs, or thesis guidelines. Search strategies, applied limits, search dates, and the number of records retrieved will be documented and made available as supplementary material with the final guideline report.

Institutional guidelines and policy documents explicitly describing the structure or evaluation criteria for medical theses, as well as relevant methodological literature, will be included when available in English or Spanish. Documents will be eligible when they describe required thesis sections, reporting elements, evaluation criteria, methodological requirements, or ethical reporting requirements. Records limited to administrative procedures, documents unrelated to medical or health sciences training, duplicate records, and records without accessible full text will be excluded. Spanish-language documents will be reviewed in their original language, and extracted elements will be translated into English only when needed for synthesis, reporting, or item development. Selection will be performed independently by two reviewers (CRL and GLO) in two sequential stages: first, screening of titles, abstracts, or institutional descriptions to assess initial relevance; second, full-text review for inclusion. Disagreements will be resolved by consensus and, if necessary, by consultation with a third reviewer (LFRH). To reduce bias related to institutional availability, the open search will be supplemented by a targeted search by geographic region and training program type, incorporating public and private institutions whenever information is available. When institutional documents are not identified through open searches, the team will search official university or medical school websites directly and will document the absence of publicly available guidance when no eligible document is found. The number of documents identified, excluded, and finally included will be recorded, along with the country or region of the corresponding institutions. The selection process will be documented using a PRISMA-style flow diagram adapted to the different sources included in the search. Search records, eligibility decisions, reasons for exclusion, and extraction matrices will be maintained by the research team.

In the second stage, structural and content elements will be extracted independently using a standardized matrix (institution, level of training, document type and year, required sections, methodological requirements, and ethical standards). Discrepancies will be resolved through adjudication. Initial agreement between reviewers will be documented for each selection stage. The kappa coefficient, which estimates the level of agreement beyond what would be expected by chance and helps determine whether the selection process is being applied consistently, will be calculated when both reviewers have independently assessed the same set of full-text documents, provided that the distribution of decisions allows for a meaningful estimate. The extracted elements will be synthesized in a thematic matrix to generate the preliminary list of items, with explicit traceability to their sources. No formal quality appraisal will be undertaken because the review is intended to identify structural components and stated reporting criteria rather than to assess the methodological quality of the source documents. The analysis will focus on identifying and comparing the structural components and stated criteria in the documents included.

#### Generation of preliminary items.

Based on the literature review, the research team will generate an initial list of items through the following process:

Extraction: three researchers will independently extract potential elements from the reviewed documents.
Consolidation: the extracted elements will be consolidated by removing duplicates and grouping similar concepts.
Categorization: the items will be organized according to the typical sections of a thesis (e.g., introduction, problem statement and research question, methods, ethical considerations, results, discussion).
Initial consensus: the full team will review the consolidated list through iterative meetings until consensus is reached on the version to be submitted to the Delphi panel. This preliminary version will include operational definitions for each item and will serve as the initial input for the Delphi rounds.

The result of this process will constitute the preliminary version of the THESES-M checklist, which will be used as the initial input for the Delphi rounds.

### Phase 2: Pre-meeting activities

#### Panel selection.

Purposive sampling will be used to recruit experts from the following key groups:

Specialists in medical education
Researchers with experience in medical research, medical education research, or thesis-based research
Methodologists
Thesis supervisors (undergraduate and postgraduate)

#### Eligibility criteria.

Inclusion criteria:

a A minimum of 5 years of experience in the supervision or evaluation of medical theses.
b Participation in formal academic evaluation or research processes.
c Familiarity with scientific reporting guidelines and thesis development processes.

Exclusion criteria:

a Lack of informed consent to participate.
b Inability to participate in the Delphi process.
c Absence of verifiable experience in thesis supervision, evaluation, or methodological review.

This composition will enable the integration of complementary perspectives relevant to the development of the guideline. Recruitment will seek geographic diversity (across different regions of the country and, where feasible, other countries), institutional diversity (public and private universities), and methodological diversity (experts with experience in evaluating clinical, educational, and health systems studies). International representation will be sought through targeted invitations to experts from countries where medical theses constitute a formal component of undergraduate or postgraduate medical training, as identified through the literature review, institutional networks, and participant nominations. The process will begin with direct invitations to identified experts and will be complemented by participant nominations to identify colleagues whose profiles are not yet represented. The final composition of the panel will be documented.

### Participants

Approximately 30 experts will be recruited (target range: 28–32). This range is consistent with the guidance and practice described by Moher et al., whose framework for reporting guideline development has employed iterative Delphi processes with panels ranging from small expert groups to over 100 participants depending on the scope and representativeness required [16]. For THESES-M, the target range was defined to ensure representation across the four predefined key groups, with approximately 7–8 participants per group at recruitment, and to preserve this diversity across three Delphi rounds. This initial range also provides a buffer for expected attrition while maintaining operational feasibility. Response rates for each round and, where applicable, the attrition rate will be reported, together with the documented reasons. To limit attrition, panelists will be informed of the expected schedule at recruitment, receive reminders during each round, and have a defined response window. Non-response will be documented, and panelists will not be replaced between rounds. To manage attrition prospectively, completion of the final Delphi round by at least 20 panelists will be required for final Delphi consensus to be interpreted as sufficiently stable. If fewer than 20 panelists complete the final round, the participation pattern will be reported, the strength of Delphi consensus will be interpreted cautiously, and unresolved items will be addressed during the consensus meeting through structured discussion and voting.

### Delphi rounds

Three Delphi rounds will be conducted to assess the importance of proposed items and to establish consensus on their inclusion, exclusion, or revision [18].

### Delphi Round 1

Panelists will receive the preliminary set of items, their operational definitions, instructions, and evaluation criteria. Using a 1-to-10 Likert scale (1 = lowest importance, 10 = highest importance), they will independently rate each item's importance. The assessment will be conducted through an electronic form (Google Forms). Spaces will be provided for qualitative comments and suggestions on modifications or the inclusion of new items.

### Importance categories

For operational purposes, Likert scores will be grouped into four importance categories, following methodological precedents used in consensus studies [19–21]. The 10-point scale was selected to allow finer discrimination of perceived item importance during the initial rating process. This classification will be used solely to facilitate score interpretation and item handling during the Delphi rounds. Consensus decisions will remain based on the predefined percentage thresholds, supported by complementary measures of dispersion, rather than on the categories alone.

- 1–4: low importance (the item should not be included)
- 5–6: moderate importance (its inclusion could be considered)
- 7–8: high importance (likely to be included)
- 9–10: very high importance (essential for inclusion)

### Consensus criteria

An item will be considered to have reached consensus when any of the following criteria are met:

- Consensus for inclusion: ≥ 80% of panelists rate the item ≥7, combining the categories of high importance (7–8) and very high importance (9–10).
- Consensus for exclusion: ≥ 80% of panelists rate the item ≤4, corresponding to the category of low importance.
- No consensus: neither of the above criteria is met.

### Item handling according to consensus status

- Items reaching consensus for inclusion (≥80% rated ≥7): will be included in the preliminary version of the checklist and will not be re-evaluated in subsequent rounds.
- Items reaching consensus for exclusion (≥80% rated ≤4): will be proposed for definitive exclusion.
- Items without consensus: will be retained for re-evaluation in subsequent rounds. Those reaching 70–79% agreement will be considered near-consensus items and will not be automatically discarded. Items with a predominance of moderate-importance ratings (5–6), or with dispersed ratings that do not meet the criteria for inclusion or exclusion, will be classified as items without consensus. Near-consensus items and items with moderate or dispersed ratings will not be included or excluded on the basis of these ratings alone. If disagreement persists after the third round, they will be discussed and resolved by structured voting during the consensus meeting.

### Additional measures

The median and interquartile range (IQR) of the scores will be calculated for each item. This will allow quantification of the degree of dispersion in the ratings and provide structured feedback to panelists in subsequent rounds. An IQR ≤ 2 will be interpreted as low dispersion and will be used as a complementary indicator to support the assessment of consensus. For items evaluated in more than one round, absolute and percentage changes in IQR between consecutive rounds will also be documented and interpreted as descriptive indicators of stability in rating dispersion.

Items may be reworded between rounds if qualitative comments consistently indicate ambiguity or lack of clarity. All modifications will be documented in the feedback report for the subsequent round, specifying the original version, the rationale for the change, and the revised version. Panelists may suggest new items; those proposed independently by at least three panelists from different groups will be included for evaluation in the next round.

Qualitative comments from each round will be analyzed through thematic coding by two investigators independently, identifying patterns of ambiguity, suggestions for rewording, or proposals for new items. Coding will begin with these three predefined categories, and new categories may be added when comments do not fit them. After independent coding, both investigators will compare classifications, resolve discrepancies by consensus, and agree on a final coding matrix.

Consensus stability across rounds will be assessed by calculating Kendall’s coefficient of concordance (W) for items evaluated in multiple rounds, along with changes in IQR. Kendall’s W, which summarizes the degree of concordance among panelists when rating the same set of items and helps describe whether agreement is stable across Delphi rounds, will be used as a measure of agreement. Values ≥0.8 will be interpreted as very strong concordance, but lower values will not by themselves determine item decisions. Item decisions will remain based on the predefined consensus thresholds. The percentage of items whose consensus classification changes between consecutive rounds will be documented as an indicator of the deliberative process's evolution.

### Delphi Round 2

Items that do not reach consensus in the first round, or that are modified on the basis of qualitative comments, will be resubmitted for a second evaluation. Panelists will receive an anonymized summary including the distribution of scores from the previous round (median, interquartile range, and frequencies), a synthesis of qualitative comments grouped thematically, and their own previous rating. This feedback will allow panelists to review their assessment in relation to the group response while preserving independent judgment. Individual ratings from other panelists will not be disclosed, and qualitative comments will be presented in aggregated form to reduce the influence of identifiable responses. The same 1-to-10 scale, importance categories, and consensus criteria defined for the first round will be used. Items that remain without consensus will proceed to the third Delphi round.

### Delphi Round 3

In the third round, items that have not reached consensus in the previous rounds will be re-evaluated. Panelists will receive an updated summary of the scores and comments accumulated across the first two rounds. The same scale, categories, and consensus criteria will be maintained. Items that remain without consensus will be reserved for resolution by structured voting during the in-person or synchronous virtual consensus meeting.

### Phase 3: Consensus meeting

Consensus activities will be conducted in person at the Faculty of Medicine of the National Autonomous University of Mexico, with synchronous remote participation enabled if required for panelists unable to attend on-site. During the meeting, the results of previous activities will be presented, including summaries of the Delphi rounds and the relevant evidence used to develop the items.

The meeting will be facilitated by members of the research team, who will present unresolved items, support structured discussion, and coordinate voting procedures when consensus has not been reached during the Delphi rounds.

The activities will include:

- Presentation of the results of the Delphi rounds.
- Analysis of the methodological justification for the included items.
- Review of the proposed order and the thematic relationship between sections.
- Definition of a flow diagram for presentation of the checklist.
- Agreements on responsibilities for drafting the explanation and elaboration document.

The outcome of the meeting will be a consolidated version of the checklist and a detailed work plan for the development of the explanation and elaboration document and dissemination materials.

### Outcomes

The primary outcome of this study is the THESES-M checklist: a consensus-based set of reporting items for medical theses, defined as those items for which ≥80% of panelists assign a score of ≥7 on a 1-to-10 Likert scale across the Delphi rounds and confirmed during the consensus meeting. The checklist will be produced at the end of Phase 3 and consolidated during Phase 4.

The secondary outcome is the explanation and elaboration document, which constitutes the instrument supporting the checklist. For each item, it will provide a definition, a rationale for its inclusion, and annotated examples to guide its interpretation and application. Its clarity, comprehensibility, and feasibility will be evaluated during pilot testing (Phase 4) through a structured questionnaire administered to participating students.

### Phase 4: Post-meeting activities

Following the consensus meeting, the team will integrate the agreements reached to produce the checklist version derived from the consensus process. This version will incorporate the items accepted during the Delphi rounds and the editorial adjustments defined during the meeting. Its purpose will be to structure the document that will be evaluated through pilot testing.

### Development of the explanation and elaboration document

The team will prepare the explanation and elaboration document, incorporating definitions, clarifications, and examples to facilitate the interpretation of each item. Consistency between the checklist and the content of the explanation and elaboration document will be reviewed to ensure conceptual precision and internal coherence.

### Pilot testing

The checklist and the explanation and elaboration document resulting from the consensus process will undergo pilot testing with the following aims: (a) to assess the clarity of the items and their operational definitions; (b) to verify that the explanations make it possible to understand what information should be reported to satisfy each item; (c) to estimate the time required to complete the checklist; (d) to identify feasibility barriers in its application; and (e) to explore its perceived usefulness and acceptability as a reporting tool. This preliminary evaluation will provide information on the instrument's usability in real-world conditions.

Approximately 20–25 students will participate in pilot testing. Inclusion criteria will comprise students currently enrolled in undergraduate, residency, master’s, or doctoral medical training who have completed data collection and analysis and are in the thesis-writing stage. Students will be recruited through purposive sampling to include different levels of medical training and methodological approaches. Participation in pilot testing will be voluntary, and use of THESES-M during the pilot will not be mandatory for thesis approval, degree completion, or any other academic requirement. Each participant will receive the checklist together with the explanation and elaboration document and will apply both to their own thesis. For each item, they will assess: (a) whether the wording is clear; (b) whether the explanation provided allows them to identify what information should be reported; (c) whether it is feasible to apply it to their type of study; and (d) suggestions for improvement. In addition, they will complete a structured questionnaire including time spent applying the tool, difficulties encountered, perceived usefulness and acceptability of the explanation and elaboration document, and practical barriers to implementation. Feasibility will therefore be assessed through application time, perceived difficulty, applicability to different thesis study designs, clarity of instructions, perceived usefulness, acceptability, and practical barriers to implementation. The questionnaire will include 5-point Likert-scale items and open-ended questions. Before use, it will undergo content and clarity validation by members of the research team and a small group of experts in methodology and medical education. This process will assess its relevance to the pilot aims, wording, and coverage of the intended domains.

The observations will be systematized through descriptive and thematic content analyses to identify recurring patterns of ambiguity or comprehension problems.

### Data management plan

Data generated during the Delphi rounds and pilot testing will be stored in de-identified form in password-protected institutional files accessible only to the research team. Any information required for participant contact or follow-up will be stored separately from response data. Only aggregated results will be reported, and access to study data will be subject to ethical and confidentiality restrictions. The de-identified dataset and supporting documentation will be made available through the OSF repository after study completion, provided that no information compromises participant confidentiality or allows individual participants to be identified.

### Safety considerations

This study does not involve clinical interventions, biological samples, or procedures that may pose risks to participants. Safety considerations are therefore not applicable.

### Implementation materials

Templates and support resources will be prepared to facilitate institutional adoption of THESES-M, including brief guides and instructions for use by students and thesis supervisors.

### Final consolidation

The team will integrate the agreements reached during the consensus process and the feedback obtained during pilot testing to produce the final version of the checklist and the explanation and elaboration document. The THESES-M checklist and its explanation and elaboration document will be submitted for inclusion in the EQUATOR Network and published with open access to support international dissemination.

### Ethics

Ethical approval was obtained from the Research Committee and the Research Ethics Committee of the Faculty of Medicine of the National Autonomous University of Mexico (FM/DI/068/2025). Participation in the Delphi rounds and pilot testing will be voluntary, and all participants will provide written informed consent prior to their inclusion. Data will be treated in accordance with confidentiality standards; responses will be anonymized during analysis and only aggregated results will be reported.

### Study status and timeline

**Fig 1:**
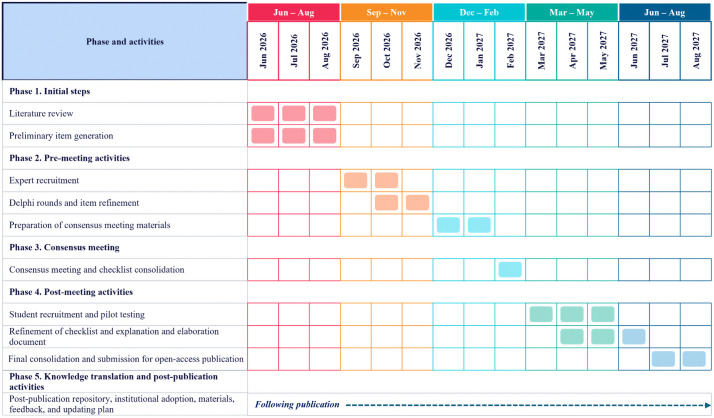
Anticipated study timeline for the development of THESES-M.

At the time of manuscript submission, the study is in the protocol phase. The literature review and preliminary item generation (Phase 1) are scheduled from 1 June 2026–31 August 2026. Experts for the Delphi panel will be prospectively recruited between 1 September 2026 and 31 October 2026. Distribution of materials, Delphi rounds, and item refinement (Phase 2) will begin after panelists have confirmed participation and will be conducted between October and November 2026. Preparation of consensus meeting materials, including the summary of Delphi results and unresolved items, will be conducted between 1 December 2026 and 31 January 2027. The consensus meeting (Phase 3) is scheduled between 1 February 2027 and 28 February 2027. Student recruitment and pilot testing will be conducted between 1 March 2027 and 31 May 2027, followed by refinement of the checklist and the explanation and elaboration document between 1 April 2027 and 30 June 2027 (Phase 4). All participants, including Delphi panel experts and students involved in pilot testing, will provide written informed consent, obtained electronically through an online form prior to participation. Participants will only gain access to the study materials after providing consent. Participation will be voluntary and response data will be stored and analyzed in de-identified form. No minors will be included in this study. Final consolidation and submission for open-access publication are scheduled between 1 July 2027 and 31 August 2027. Phase 5 activities will begin following publication of THESES-M. The anticipated study timeline is summarized in Fig 1.

### Phase 5: Knowledge translation and post-publication activities

Following publication of THESES-M, knowledge translation and dissemination activities will be established to ensure accessibility, appropriate use, and ongoing updating of the guideline. A dedicated digital repository will be set up to host the checklist, the explanation and elaboration document, implementation materials, and version records. Its purpose will be to provide a single point of access and facilitate open availability.

The team will promote its availability to academic programs, medical schools, and university networks responsible for research training to facilitate its integration into thesis supervision, evaluation, and reporting processes. Support materials—such as templates and annotated examples—will also be developed to facilitate interpretation of the checklist across different levels of training.

Official translations will also be developed through a translation/back-translation process and will be published in the repository with their corresponding version records. The repository will include a mechanism for receiving user feedback, enabling the identification of interpretive difficulties and areas requiring adjustment. In parallel, the first experiences of institutional implementation will be documented to identify patterns of use and potential critical issues.

**Fig 2:**
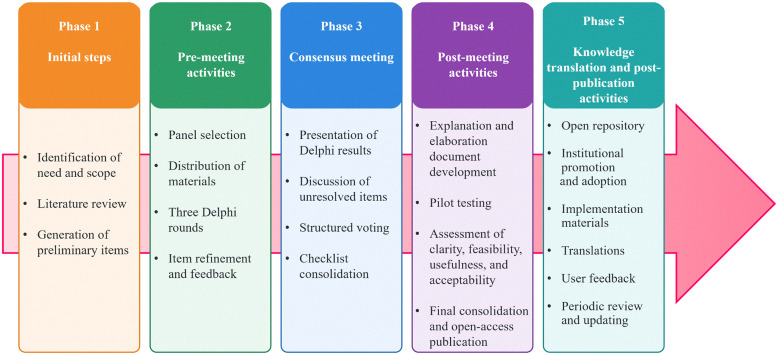
Overview of the five-phase THESES-M development process.

Finally, a process of periodic review will be established to incorporate new evidence, clarify ambiguous elements, and update the content to address identified needs. All modifications will be recorded in the repository to ensure traceability and transparency. Fig 2 summarizes the five-phase development process of THESES-M, from initial steps to knowledge translation and post-publication activities.

### Artificial Intelligence (AI) use declaration

OpenAI ChatGPT was used to support drafting, language editing, wording refinement, and organization of the manuscript. All outputs were reviewed, edited, and validated by the authors. The tool was not used to generate study data, conduct analyses, identify, select, or generate references, or replace the authors’ methodological decisions or interpretations. The authors take full responsibility for the final content of the manuscript.

## Discussion

THESES-M seeks to address a documented gap in the academic infrastructure for reporting medical theses [9]. Although reporting guidelines have transformed scientific communication across multiple fields [15,22,23], no equivalent standards currently exist for medical theses as academic documents. By establishing explicit reporting criteria, this project aims to provide a structured framework to support more consistent evaluation and reporting of medical theses.

The proposed methodology combines literature review, Delphi consensus rounds, a consensus meeting, and pilot testing to support the iterative development of the guideline [16–19]. This process is intended to refine the proposed items through the integration of complementary perspectives from medical educators, researchers, methodologists, and thesis supervisors, thereby strengthening the conceptual clarity, relevance, and usability of the final checklist and its explanation and elaboration document.

THESES-M may contribute to more explicit reporting practices in medical education from early formative stages. A transparent and clearly defined reporting structure does more than organize information; it also clarifies the study's internal logic and situates the student’s work within recognized scientific conventions [3,9]. A structured reporting framework may facilitate clearer academic evaluation and more consistent communication of these documents. At the same time, adherence to a reporting guideline does not in itself guarantee methodological quality; rather, it supports more transparent and evaluable reporting. Its usefulness will depend on factors such as institutional adoption, user adherence, and the characteristics of the training environments in which it is implemented.

Transparent reporting is also relevant from an ethical perspective. Its absence limits critical appraisal, weakens reproducibility, and reduces the usability of academic work [24–26]. By defining minimum standards of clarity, completeness, and explicit reporting, THESES-M aligns with principles recognized by the EQUATOR Network and extends their application to the field of research training in medicine [15,23,26].

The implementation of a reporting guideline for medical theses may also encounter institutional and cultural resistance. Standards may be perceived as limiting methodological flexibility or as introducing forms of homogenization that do not fully account for disciplinary variation or differences across training contexts. Institutions with established traditions in thesis supervision and evaluation may also regard external guidance as interference with existing academic practices. These concerns require explicit recognition, because the value of a reporting guideline lies in making research communication more explicit and comparable, not in prescribing a single methodological model. In this sense, THESES-M is intended to operate at the level of reporting rather than at the level of methodological choice.

This study has limitations that should be acknowledged. The composition of the expert panel may influence the prioritization of items included in the final guideline, despite efforts to ensure disciplinary, methodological, institutional, and geographic diversity. In addition, the identification of institutional guidance documents may depend on their public availability, which could restrict the range of materials included in the literature review. This limitation will be mitigated by combining open searches with targeted searches of institutional websites by geographic region and training program type, and by documenting cases in which no publicly available guidance is found. Pilot testing will be conducted with students who are already in the thesis-writing stage, so the evaluation will primarily reflect usability at that phase rather than across the entire research process. Initial pilot testing will also be conducted within one institutional context; therefore, subsequent use in other educational settings may provide additional information on feasibility and implementation.

Any substantial modifications to the study procedures after protocol publication will be documented and reported in subsequent outputs arising from the study.

Overall, THESES-M seeks to establish minimum standards for the reporting of medical theses. By specifying the essential sections and content that should be included in these documents, the guideline is intended to provide a structured basis for clearer and more explicit academic and scientific communication. Its implementation, institutional uptake, and effects on reporting practices will need to be evaluated in subsequent studies.
