# Supplementary material for: THESES-M: protocol for the development of a reporting guideline for medical theses
Source: PLoS One. 2026 Aug 7;21(8):e0354899. doi: 10.1371/journal.pone.0354899 (PMC13450800; doi:10.1371/journal.pone.0354899)
Supplement: Supplementary file 2 [file pone.0354899.s004.pdf]

Response to reviewers

We sincerely thank all reviewers for the time, expertise, and care dedicated to evaluating our manuscript. Their thoughtful comments and suggestions helped us improve the clarity, methodological transparency, and overall presentation of the protocol. We appreciate their constructive feedback and have addressed the comments to the best of our ability in the revised manuscript.

Reviewer #1:

This manuscript presents a structured and methodologically sound protocol for developing the THESES-M reporting guideline, addressing the important gap of standardized reporting criteria in medical theses. The rationale is well-supported by relevant literature.

The methodology adheres to EQUATOR Network recommendations and includes a systematic literature review, Delphi rounds, predefined consensus criteria, and a structured consensus meeting, enhancing its robustness. Pilot testing ensures the checklist's feasibility and usability.

The methodology is detailed and reproducible, with transparent processes for literature review, item generation, expert selection, Delphi procedures, and data analysis. The combination of quantitative measures (median, IQR, Kendall's W) and qualitative thematic analysis strengthens the consensus validity.

We thank the reviewer for the time spent evaluating our manuscript and for the overall assessment of the protocol. We appreciate the comments on the rationale, methodological structure, and development process of THESES-M. The specific observations raised by the reviewer are addressed below.

However, some areas need clarification:

1. Data Availability: Specify the repository for de-identified data to enhance transparency.

Thank you for this observation. We have specified that the de-identified data derived from the Delphi rounds and pilot testing will be deposited in the Open Science Framework after study completion. We also retained the ethical and confidentiality restrictions applicable to the study data, given the potential identifiability of Delphi panelists and pilot participants in some materials (lines 16–19 new version).

2. Literature Review Scope: Include more detail on inclusion/exclusion criteria and quality appraisal.

Thank you for this comment. We revised the literature review section to specify the eligibility and exclusion criteria more clearly (lines 144–147, new version). We also clarified that no formal quality appraisal will be undertaken because the review is intended to identify structural components and stated reporting criteria rather than to assess the methodological quality of the source documents (lines 172–174, new version).

3. Expert Panel Diversity: Clarify how international representation beyond Mexico will be achieved.

Thank you for this comment. We revised the panel selection section to clarify how international representation will be sought. We added that experts from countries where medical theses constitute a formal component of medical training will be identified through the literature review, institutional networks, and participant nominations (lines 213–216, new version).

4. Potential Bias: Discuss strategies to mitigate selection bias from relying on publicly available documents.

Thank you for this comment. We revised the literature review and limitations sections to clarify how selection bias related to public availability will be addressed. We specified that open searches will be complemented by targeted searches of official university or medical school websites by geographic region and training program type (lines 153–158, new version). We also added that cases in which no publicly available guidance is found will be documented (lines 499–501, new version).

5. Minor Language Edits: Polishing some sections could improve readability.

Thank you for this comment. We reviewed the manuscript and made targeted language edits in the Abstract, Methods, and Discussion to improve readability.

Overall, this is a high-quality protocol following established standards and has potential contributions to medical education and research reporting. With minor revisions, it would be suitable for publication.

We thank the reviewer for the positive assessment of the protocol and for the constructive comments. We have addressed each point below and made targeted revisions to improve the clarity of the manuscript.

Reviewer #2:

Rationale: The manuscript explains the gap (the absence of uniform reporting standards for medical theses) and explains why THESES-M is necessary. The reasoning is backed by relevant literature that shows variations in thesis structure, low publication conversion rates, and faults noted by supervisors. The goal is clearly stated: to create a reporting guideline utilizing proven EQUATOR Network techniques.

Although the protocol is generally sound, there are a few areas that need to be strengthened or clarified before I would say it is completely robust:
We sincerely appreciate the reviewer's time and careful reading of our manuscript. We are grateful for the positive assessment of the protocol and for the comments regarding its rationale, methodological structure, and development process. We address each observation below.

The majority of methodological literature uses >0.5–0.7, although the Delphi consensus level for stability (Kendall's W ≥ 0.8) is exceptionally strict. This needs to be clarified or updated.
We appreciate this observation. We revised the text to clarify the role of Kendall's W in the Delphi analysis. Kendall's W will be used as a descriptive measure of agreement. Values ≥0.8 will be interpreted as very strong concordance, but lower values will not by themselves determine item decisions. Item decisions will remain based on the predefined consensus thresholds (lines 297–299, new version).

It is unclear how to handle "moderate importance" elements (scoring 5–6); clear guidelines are required.
We appreciate this observation. We revised the Delphi procedures to clarify how moderate-importance ratings will be handled. Items with a predominance of scores of 5–6, or with dispersed ratings that do not meet the predefined criteria for inclusion or exclusion, will be classified as items without consensus. They will be retained for re-evaluation in subsequent rounds and, if disagreement persists, discussed during the consensus meeting (lines 270–275, new version).

For the three rounds of the Delphi panel, no attrition management strategy is outlined.
We appreciate this observation. We revised the Participants section to specify how attrition will be managed during the Delphi process. Panelists will be informed of the expected schedule at recruitment, receive reminders during each round, and have a defined response window. Non-response will be documented, and panelists will not be replaced between rounds (lines 228–235, new version).

The qualitative analysis of comments (e.g., coding scheme, inter-rater reliability targets) is stated but not explained.

We appreciate this observation. We revised the Delphi analysis section to clarify the coding process for qualitative comments. Coding will begin with predefined categories related to ambiguity, rewording, and proposed new items. We also specified that both investigators will compare their classifications and agree on a final coding matrix (lines 290–292, new version).

These problems should be fixed to avoid unreported flexibility, but they do not render the protocol illegal.

We agree that these methodological points required clarification. We addressed them in the revised manuscript to make the planned Delphi procedures more explicit and reduce ambiguity.

Methodology: The technique is sufficiently detailed for replication and adheres to the Moher et al. framework:

We thank the reviewer for this comment.

The literature review search strategy is devoid of precise search terms, time constraints, and a methodical approach to locating institutional repositories. Replicability is so limited.

We appreciate this observation. We revised the literature review section to make the search strategy more explicit. We specified the English and Spanish search terms, the temporal limit of the search, and the approach for locating institutional repositories and guidance documents through official university and medical school websites (lines 132–141, new version).

There is insufficient information about the pilot testing questionnaire (e.g., scales, validation).

We appreciate this observation. We revised the pilot testing section to clarify that the questionnaire will include 5-point Likert-scale items and open-ended questions. We also specified that, before use, it will undergo content and clarity validation by members of the research team and a small group of experts in methodology and medical education, focusing on relevance to the pilot aims, wording, and coverage of the intended domains (lines 374–380, new version).

There is no discussion of how to handle institutional guidelines that are not in English.

We appreciate this observation. We revised the literature review section to clarify that institutional guidance available in English or Spanish will be eligible. Spanish-language documents will be reviewed in their original language, and extracted elements will be translated into English only when needed for synthesis, reporting, or item development (lines 143–145 and 147–149, new version).

The Delphi panel's sample size rationale (28–32 experts) is descriptive rather than empirically supported. Replicability would be significantly enhanced by fixing these omissions.

We appreciate this observation. We revised the Participants section to move beyond a descriptive justification and link the planned panel size more directly to the structure of the Delphi process. The revised rationale now explains how the initial target supports expert-group representation, continuity across rounds, and attrition management (lines 223-227, new version).

The work employs standard scientific English and is arranged and written clearly. Throughout, the language is understandable.

We thank the reviewer for this positive comment.

As a Reviewer, i believe Sample size justification for the Delphi panel needs strengthening. Literature review methodology lacks search strings and a systematic approach to institutional repositories. Also consensus thresholds (Kendall's $W \geq 0.8$) are too stringent; handling of scores 5–6 is ambiguous. Pilot testing design not mentioned appropriately.

We thank the reviewer for these comments. They have been addressed point by point in the revised version of the manuscript.

Conflict of interest/disclosure is missing, qualitative analysis plan, patient/public involvement rationale.

We thank the reviewer for this observation. The manuscript already included a Competing interests statement (lines 14–15, new version), which was retained in the revised version. We also clarified the qualitative analysis plan for open comments and added a Patient and public involvement statement (lines 20–22, new version).

Reference style is truncated (page 27).

We reviewed and corrected the formatting of the reference list to avoid irregular spacing, line breaks, or apparent truncation in the final pages of the manuscript.

Data availability statement does not comply with PLOS ONE policy (needs repository and timing clarification).

We revised the Data availability statement to specify that de-identified data derived from the Delphi rounds and pilot testing will be deposited in the Open Science Framework and made publicly

available upon completion of the study, subject to ethical and confidentiality restrictions (lines 16-19, new version).

I recommend minor revision.

We thank the reviewer for the recommendation and for the comments provided to improve the manuscript.

Reviewer #3:

1. Delphi Method - the protocol sets a threshold of more than 80% consensus. my recommendation is please define a clear "gray zone" management strategy. If an item receives 70–79% agreement, will it be automatically discarded, or will it be discussed in a virtual meeting? Defining the process for near-consensus items will prevent the loss of potentially valuable reporting standards.

We thank the reviewer for this observation. We revised the Delphi procedures to clarify that items reaching 70–79% agreement will be considered near-consensus items and will not be automatically discarded. These items will be retained for re-evaluation in subsequent rounds and, if disagreement persists after the third round, discussed during the consensus meeting (lines 268-273, new version).

2. Please specify the metrics for "feasibility." Will this be measured by the time it takes to complete the checklist, or by inter-rater reliability scores when two different evaluators use THESES-M to grade the same thesis? Strengthening the description of the pilot phase will provide more confidence in the guideline's eventual validation.

We thank the reviewer for this observation. We revised the pilot-testing section to specify the feasibility metrics, including application time, perceived difficulty, applicability to different thesis study designs, clarity of instructions, perceived usefulness, acceptability, and practical barriers to implementation (lines 374–380, new version). Inter-rater reliability will not be assessed in this pilot phase, which is focused on initial usability and feasibility.

3. LR - Please Include a "Knowledge Translation" or "Dissemination" section in the protocol. This could involve plans to develop an Explanation and Elaboration (E&E) document (similar to CONSORT or PRISMA) or strategies to partner with specific medical university networks to mandate the guideline's use for graduation.

We thank the reviewer for this suggestion. The protocol already included several post-publication activities, including the explanation and elaboration document (lines 400–402, new version),

implementation materials, an open repository, translations, user feedback mechanisms, and periodic updating (lines 432–448, new version). We revised the heading and wording of Phase 5 to make its knowledge translation and dissemination purpose explicit, and added medical schools and university networks as intended dissemination partners (lines 431–432 and 437–438, new version).

4. Please define a clear "gray zone" management strategy. If an item receives 70–79% agreement, will it be automatically discarded, or will it be discussed in a virtual meeting? Defining the process for near-consensus items will prevent the loss of potentially valuable reporting standards.

We thank the reviewer for this comment. This point was addressed in our response to the first Delphi-related comment, where we clarified the handling of near-consensus items reaching 70–79% agreement (lines 268-273, new version).

5. Please define an IQR ≤ 2 as the objective cutoff for consensus.

We thank the reviewer for this suggestion. We revised the Delphi analysis section to specify that an IQR ≤2 will be interpreted as low dispersion and used as a complementary indicator to support the assessment of consensus (lines 279-280, new version).

6. please measured dispersion (IQR) - Low IQR - please indicates the most experts ranting are clustered closed to gather (High consensus), High IQR - Please indicates that experts opinions are widely spread in low consensus.

We thank the reviewer for this comment. We clarified that the IQR will be used to assess dispersion in expert ratings. We also specified that, for items evaluated in more than one round, absolute and percentage changes in IQR between consecutive rounds will be documented as descriptive indicators of stability in rating dispersion (lines 280-282, new version).

7. Please add "change in IQR' alongside kendalls W to confirm when the the panel's opinion has stabilized

We thank the reviewer for this helpful suggestion. We incorporated this point into the Delphi analysis by considering both agreement among panelists and changes in the dispersion of their ratings across rounds. This will help describe whether panel ratings become more stable across rounds (lines 293-296, new version).

8. Please adding the percentage change in the IQR between rounds. If the IQR does not change significantly between Round 2 and Round 3, it proves "stability," meaning further rounds would not change the outcome. This adds a second layer of statistical rigor to your Delphi process.

We thank the reviewer for this helpful suggestion. We incorporated absolute and percentage changes in IQR between consecutive rounds as descriptive indicators of stability in rating dispersion. These changes will provide an additional descriptive element to assess whether expert ratings become more stable across rounds (lines 280–282, new version).

9. please polishing the novelist

We thank the reviewer for this comment and for the helpful suggestions provided throughout the review process. We carefully reviewed the manuscript and made targeted edits where needed.

Reviewer #4:

The manuscript is a study protocol describing the development of a guideline framework, THESES-M, with the intent to provide minimal standard requirements on consistency and evaluation of medical theses. The authors had identified this gap through the variability of how medical theses are evaluated and, especially, on the observation around the variability in how medical theses are structured, evaluated, and ultimately translated into scientific publications. The authors make the case that inconsistency in thesis quality may contribute to the relatively low rate of conversion into manuscripts, and their effort to develop a standardized framework is both timely and relevant. The strength of the work lies in its clear identification of this gap, as well as in the overall structured and replicable protocol presented. The inclusion of pilot testing with students is particularly valuable, as it grounds the framework in practical application and usability.

We sincerely thank the reviewer for this generous and constructive assessment. We appreciate the recognition of the relevance of the protocol and of the value of including pilot testing with students. The comments helped us identify areas where the manuscript could be clearer and more useful to readers.

Several areas would benefit from greater clarity and refinement to strengthen the manuscript's overall impact and generalizability. The scope and context of the work could be more explicitly framed at the outset. Because medical theses are not a universal requirement, it would be helpful for the introduction to clearly state upfront where and at what levels of medical education (e.g., undergraduate, graduate, residency) such theses are typically required – particularly in regions such as Europe, Asia, Africa, and Latin America. Relatedly, the authors should clarify how the proposed framework may be applicable, or adaptable, in settings where medical theses are not a standard

requirement. Additional clarity and supporting context are also needed for the claim that master's theses have low rates of conversion into publications.

We thank the reviewer for this helpful observation. We revised the first paragraph of the Introduction to clarify that medical theses are not mandatory in all medical training systems, while specifying the settings, regions, and levels of training in which they remain relevant (lines 48–52, new version). This framing keeps the scope of THESES-M focused on contexts where medical theses form part of medical training. We also added supporting context on thesis-to-publication rates, including medical student master's theses (lines 63–65, new version).

Methodologically, the protocol is generally clear and well organized, but additional detail would improve transparency and reproducibility. For example, a brief explanation of the Delphi consensus process, including who participates, how meetings are facilitated, and how consensus is defined and managed if not reached, would strengthen the rigor of the approach. The rationale for providing participants with prior rankings should also be explained, along with any steps taken to mitigate potential bias across Delphi rounds. Similarly, the pilot study would benefit from clarification regarding the selection of the 20–25 students, whether participation to use the THESES-M guidelines was voluntary or mandatory, how consent was obtained, and how potential self-selection bias was considered. That is, were all eligible students required to use the THESES-M guidelines and were asked to volunteer to complete the survey?

We thank the reviewer for this helpful comment. We revised the Methods to provide further detail on the Delphi and pilot-testing procedures. Specifically, we clarified the composition and eligibility criteria of the Delphi panel (lines 193–208, new version), the target panel size and procedures to monitor and manage attrition (lines 223–235, new version), and the role of the research team in facilitating structured discussion and voting for unresolved items (lines 323–325, new version). We also explained the rationale for providing anonymized feedback between rounds and the measures used to preserve independent judgment and reduce the influence of identifiable responses (lines 306–309, new version). Finally, we clarified that students in the pilot test will be selected through purposive sampling, that participation will be voluntary, and that use of THESES-M will not be mandatory for thesis approval or degree completion (lines 364–368, new version).

Additional clarity in several areas is recommended:

1. The manuscript would benefit from clearer operational definitions, including concrete examples (e.g., what constitutes a "medical thesis"), and more specificity regarding purposive sampling (e.g., types of researchers and definitions of undergraduate versus postgraduate in this context).

We thank the reviewer for this helpful suggestion and for the opportunity to expand this information. We refined this section by adding definitions of undergraduate and postgraduate training in the context of this study and the operational definition of medical theses provided in the same section (lines 91–98, new version). We also expanded the description of purposive sampling, which outlines the predefined expert groups, eligibility criteria, and intended geographic, institutional, and methodological diversity of the panel (lines 193–213, new version), by specifying that international representation will be sought through targeted invitations to experts from countries where medical theses are a formal component of undergraduate or postgraduate medical training (lines 213–216, new version). In addition, we clarified that the target panel size was defined to ensure representation across the four predefined key groups (lines 223–227, new version).

2. The literature review process should specify the systematic procedures used (such as PRISMA), and the authors should clarify how documentation and data management will occur.

We thank the reviewer for this helpful suggestion. We expanded the literature review procedures by specifying the databases, institutional sources, search terms, dates, limits, and documentation of retrieved records (lines 132–141, new version). We also clarified eligibility criteria, exclusion criteria, language handling, and extraction procedures (lines 143–149, new version). In addition, we specified that the selection process will be documented using a PRISMA-style flow diagram and that search records, eligibility decisions, reasons for exclusion, and extraction matrices will be maintained by the research team (lines 159–162, new version).

3. Additionally, the decision not to conduct a formal quality appraisal warrants justification.

We thank the reviewer for this helpful comment. We added a justification for not undertaking a formal quality appraisal, clarifying that the review is intended to identify structural components and stated reporting criteria rather than to assess the methodological quality of the source documents (lines 172–174, new version).

4. Providing brief explanations of the statistical measures used, such as kappa and Kendall's coefficients, would also improve accessibility for a broader audience.

Thank you for this suggestion. We added brief explanations of the kappa coefficient (lines 167-168) and Kendall's W (lines 294–296, new version) to clarify what each measure contributes to the analysis.

5. The inclusion of a visual summary (e.g., a figure or flow diagram) of the protocol would help readers more readily grasp the overall process. Finally, while the framework is promising, it is

currently limited to a single institutional context, and this should be acknowledged more explicitly as a limitation in terms of generalizability.

We thank the reviewer for this suggestion. We added a flow diagram to help readers follow the overall development process of THESES-M (Figure 2). We also expanded the limitations to specify that the initial pilot testing will take place within one institutional context and that subsequent use in other educational settings may provide further information on feasibility and implementation (lines 503–505, new version).

Overall, the study is feasible and addresses a meaningful gap in the field. With improved clarity in scope, enhanced methodological transparency, and some tightening to reduce repetition, the manuscript would be well positioned to make a valuable contribution to the standardization of medical thesis development and evaluation.

We sincerely thank the reviewer for this encouraging assessment. The comments helped us identify where the protocol needed clearer framing and more precise methodological detail. We consider these revisions have strengthened the manuscript.

Reviewer #5:

General Assessment The proposed protocol, THESES-M, aims to establish a reporting standard for medical theses, designed for inter-institutional application across various disciplines within the health sciences. This is a laudable goal that aligns perfectly with the rigorous, open-science mandate of PLOS ONE. Establishing these standards will be a highly beneficial undertaking for medical supervisors, researchers, and students globally, and their eventual publication in PLOS ONE will ensure the resulting guidelines are universally available.

Overall, the protocol is methodologically robust and closely adheres to the recognised guidance for guideline development set out by Moher et al. and the EQUATOR Network. It represents a strong candidate for publication. The following minor revisions and methodological clarifications are offered primarily for the benefit of the authors to further strengthen the study's reproducibility and psychometric transparency.

We sincerely thank the reviewer for the time dedicated to evaluating our manuscript and for the thoughtful comments. We carefully considered each suggestion and revised the manuscript accordingly.

Major Comments

Protocol Timeline and PLOS ONE Compliance: It is understood from the text that data collection and the Delphi rounds have not yet commenced, which satisfies PLOS ONE's prospective requirement for study protocols. However, to ensure immediate clarity for the editorial board and future readers regarding the prospective nature of the work, presenting the anticipated study timeline as a formal table or Gantt chart is highly recommended.

We thank the reviewer for this helpful suggestion. We added anticipated dates for the remaining phases of the study to make the prospective status of the protocol clearer, including Delphi activities, the consensus meeting, pilot testing, final consolidation, and open-access publication (lines 413–420 and 425–427, new version). The anticipated study timeline is also summarized in Figure 1.

Considerations for the Likert Scale: The protocol proposes utilising a 10-point Likert-like scale to rate initial items, which is subsequently grouped into four operational categories. While there is precedent for wider scales in healthcare consensus methodologies (such as the 9-point RAND/UCLA appropriateness method), collapsing a 10-point ordinal scale into four distinct bins may introduce unnecessary variance and limit the granularity the scale was originally intended to capture. The authors might consider whether a more common 5- or 7-point scale would be more psychometrically stable for consensus thresholding. If the 10-point-to-4-bin structure is retained, a brief justification of its statistical validity and thresholding mechanism would strengthen the methodology.

We thank the reviewer for this helpful comment. We added a brief justification for the 10-point scale, indicating that it was selected to capture finer differences in perceived item importance during the initial rating process. We also clarified that the four operational categories will be used only to facilitate interpretation and item handling during the Delphi rounds. Consensus decisions will remain based on the predefined percentage thresholds and supported by dispersion measures, rather than on these categories alone (lines 247–251, new version).

Reproducibility of the Literature Review: To ensure the resulting guideline is truly applicable across diverse global institutions, the initial literature review phase should be explicitly defined in terms of its linguistic scope (e.g., clarifying whether Spanish or other non-English literature will be included). Furthermore, while a full formal scoping review may be out of scope for initial item generation, depositing the exact Boolean search strings, database limits, and search dates as a Supplementary File would greatly enhance the study's reproducibility.

We thank the reviewer for this helpful suggestion. We expanded the literature review procedures to clarify the linguistic scope of the search, specifying that documents available in English or Spanish

will be eligible and that Spanish-language documents will be reviewed in their original language (lines 143–149, new version). To strengthen reproducibility, we also added that search strategies, applied limits, search dates, and the number of records retrieved will be documented and shared as supplementary material with the final guideline report (lines 132–141, new version). In addition, the selection process will be documented using a PRISMA-style flow diagram, with eligibility decisions, reasons for exclusion, and extraction matrices recorded for transparency (lines 159–162, new version).

Delphi Panel Attrition: Longitudinal Delphi panels frequently experience attrition across multiple rounds. While the protocol defines the intended initial panel size, it would benefit from a quantified a priori coping plan based on absolute numbers. To protect the statistical validity of the final consensus, the authors are encouraged to define their minimum acceptable absolute panel size (e.g., stating that consensus will not be considered valid if the panel drops below N = 20) rather than relying solely on optimal initial recruitment numbers.

We thank the reviewer for this helpful guidance. We strengthened the Delphi procedures by adding a predefined approach to reduce and manage panel attrition. This includes practical measures to support retention across rounds and documentation of non-response. We also introduced an absolute minimum for the final Delphi round, so that consensus will only be interpreted as sufficiently stable if at least 20 panelists complete it. If this threshold is not reached, the participation pattern will be reported, the findings will be interpreted with caution, and unresolved items will proceed to structured discussion and voting during the consensus meeting (lines 228–235, new version).

Minor Comments

Data Availability: The authors state that de-identified data will be made available upon study completion, which is the correct approach for a study protocol prior to data collection. However, to ensure seamless compliance with PLOS ONE’s strict Data Availability policies, the authors should explicitly state where or how they intend to make this data available in the future (e.g., indicating that it will be deposited in a specific public repository such as OSF or Figshare, or published as Supplementary Information alongside the final manuscript).

We thank the reviewer for this helpful suggestion. We revised the Data Availability statement to make the planned repository explicit. The revised manuscript specifies that the de-identified data derived from the Delphi rounds and pilot testing will be deposited in the Open Science Framework (OSF) and made publicly available after study completion, subject to ethical and confidentiality restrictions (lines 16–19, new version).

Pilot Testing Phase: Please ensure that the pilot testing phase (Phase 4), which is mentioned in the abstract, is described with sufficient detail in the full text. Specifically, as this is likely a qualitative usability exercise, the protocol should note the target number of users anticipated to reach data saturation (e.g., 5-10 users) and detail the specific evaluation metrics (e.g., clarity, feasibility) that will be applied to the consensus-based checklist and the explanation and elaboration document.

We thank the reviewer for this thoughtful and helpful comment. The pilot-testing section describes the aims of Phase 4, including clarity, feasibility, completion time, usefulness, and acceptability (lines 355–361, new version). We further clarified the planned sample and recruitment approach, indicating that 20–25 students will be recruited through purposive sampling and that participation will be voluntary, with no academic requirement to use THESES-M during the pilot (lines 362–368, new version). We also specified the feasibility and usability metrics, the use of 5-point Likert-scale items and open-ended questions, and the planned content and clarity validation of the questionnaire before use (lines 374–380, new version).

Additional revision

We have also added an Artificial Intelligence (AI) use declaration to the manuscript, in accordance with PLOS policy. The declaration specifies the tool used, the purpose of its use, the review and validation of outputs by the authors, and the authors' responsibility for the final manuscript (lines 454–459, new version).
