# Supplementary material for: THESES-M: protocol for the development of a reporting guideline for medical theses
Source: PLoS One. 2026 Aug 7;21(8):e0354899. doi: 10.1371/journal.pone.0354899 (PMC13450800; doi:10.1371/journal.pone.0354899)
Supplement: Supplementary file 3 [file pone.0354899.s001.pdf]

PLOS One

THESES-M: protocol for the development of a reporting guideline for medical theses

--Manuscript Draft--

**Manuscript Number:**	PONE-D-26-15953
**Article Type:**	Study Protocol
**Full Title:**	THESES-M: protocol for the development of a reporting guideline for medical theses
**Short Title:**	THESES-M reporting guideline protocol
**Corresponding Author:**	Geovani López-Ortiz López-Ortiz, Ph.D.<br>Universidad Nacional Autonoma de Mexico Facultad de Medicina<br>Mexico, City, MEXICO
**Keywords:**	Medical Theses; Medical Education; Research Training; Reporting Guidelines; Academic Writing.
**Abstract:**	Introduction: Medical theses represent one of the most widely produced yet least standardized forms of health sciences research. Their structure and reporting vary substantially across institutions, programs, and regions, and no formal reporting criteria exist to guide how these documents should be produced. This variability affects the consistency and evaluation of medical theses and may influence subsequent research reporting practices. This study describes the protocol for the development of THESES-M (Transparent and Holistic Evaluation Standards for Educational Studies in Medicine), a reporting guideline for medical theses.<br>Materials and methods: THESES-M will be developed in accordance with the guidance proposed by Moher et al. and the recommendations of the EQUATOR Network. The study will include a literature review to identify structural components of medical theses, generation of a preliminary list of reporting items, and three Delphi rounds with an expert panel using a 1–10 Likert scale. Consensus thresholds are predefined (≥80% rating ≥7 for inclusion; ≥80% rating ≤4 for exclusion). Agreement and stability will be assessed using median scores, interquartile ranges, and Kendall's coefficient of concordance. Items without consensus will be discussed in an in-person consensus meeting. The study outputs will include a consensus-based checklist and an explanation and elaboration document. Both will undergo pilot testing with medical students to assess clarity, feasibility, and usability.<br>Ethics and dissemination: Ethical approval was obtained from the Research and Ethics Committees of the Faculty of Medicine, National Autonomous University of Mexico (FM/DI/068/2025). Study findings will be disseminated through peer-reviewed publications and submission of the final guideline to the EQUATOR Network. THESES-M is registered with the EQUATOR Network as a reporting guideline under development.
**Order of Authors:**	Carlos Rivero-López
	Ana Elena Limón-Rojas
	Ricardo Octavio Morales-Carmona
	Claudia Jimena Vilchis-Macedo
	Liz Hamui-Sutton
	Magali Fabiola Vega-Rodríguez
	Luisa Fernanda Romero-Henríquez
	Geovani López-Ortiz
**Additional Information:**	
**Question**	**Response**
**Financial Disclosure**<br><br>Enter a financial disclosure statement that	The author(s) received no specific funding for this work.

Powered by Editorial Manager® and ProduXion Manager® from Aries Systems Corporation

describes the sources of funding for the work included in this submission. Review the submission guidelines for detailed requirements. View published research articles from *PLOS ONE* for specific examples.<br><br>This statement is required for submission and **will appear in the published article** if the submission is accepted. Please make sure it is accurate.<br><br>**Funded studies**<br>Enter a statement with the following details:<br>• Initials of the authors who received each award<br>• Grant numbers awarded to each author<br>• The full name of each funder<br>• URL of each funder website<br>• Did the sponsors or funders play any role in the study design, data collection and analysis, decision to publish, or preparation of the manuscript?<br><br>Did you receive funding for this work?	
**Competing Interests**<br><br>Use the instructions below to enter a competing interest statement for this submission. On behalf of all authors, disclose any competing interests that could be perceived to bias this work—acknowledging all financial support and any other relevant financial or non-financial competing interests.<br><br>This statement is required for submission and **will appear in the published article** if the submission is accepted. Please make sure it is accurate and that any funding sources listed in your Funding Information later in the submission form are also declared in your Financial Disclosure statement.<br><br>View published research articles from *PLOS ONE* for specific examples.	The authors have declared that no competing interests exist.

**NO authors have competing interests**<br><br>Enter: *The authors have declared that no competing interests exist.*<br><br>**Authors with competing interests**<br><br>Enter competing interest details beginning with this statement:<br><br>*I have read the journal's policy and the authors of this manuscript have the following competing interests: [insert competing interests here]*<br><br>* typeset	
**Ethics Statement**<br><br>Enter an ethics statement for this submission. This statement is required if the study involved:<br><br>• Human participants<br>• Human specimens or tissue<br>• Vertebrate animals or cephalopods<br>• Vertebrate embryos or tissues<br>• Field research<br><br>Write "N/A" if the submission does not require an ethics statement.<br><br>General guidance is provided below. Consult the submission guidelines for detailed instructions. **Make sure that all information entered here is included in the Methods section of the manuscript.**	Ethical approval was obtained from the Research Committee and the Research Ethics Committee of the Faculty of Medicine, National Autonomous University of Mexico (approval number: FM/DI/068/2025).<br><br>The study involves human participants in the Delphi consensus process and pilot testing. Participation will be voluntary, and all participants will provide written informed consent prior to inclusion. Data will be collected and analyzed in de-identified form, and only aggregated results will be reported to ensure confidentiality.

Powered by Editorial Manager® and ProduXion Manager® from Aries Systems Corporation

**Format for specific study types**<br><br>**Human Subject Research (involving human participants and/or tissue)**<br>• Give the name of the institutional review board or ethics committee that approved the study<br>• Include the approval number and/or a statement indicating approval of this research<br>• Indicate the form of consent obtained (written/oral) or the reason that consent was not obtained (e.g. the data were analyzed anonymously)<br><br>**Animal Research (involving vertebrate animals, embryos or tissues)**<br>• Provide the name of the Institutional Animal Care and Use Committee (IACUC) or other relevant ethics board that reviewed the study protocol, and indicate whether they approved this research or granted a formal waiver of ethical approval<br>• Include an approval number if one was obtained<br>• If the study involved *non-human primates*, add *additional details* about animal welfare and steps taken to ameliorate suffering<br>• If anesthesia, euthanasia, or any kind of animal sacrifice is part of the study, include briefly which substances and/or methods were applied<br><br>**Field Research**<br><br>Include the following details if this study involves the collection of plant, animal, or other materials from a natural setting:<br>• Field permit number<br>• Name of the institution or relevant body that granted permission	
Data Availability<br><br>Before publication, Authors are required to make fully available and without restriction all data underlying their findings. Sharing the data underlying your findings increases the visibility, credibility, and impact of your work, while supporting transparency and future scientific discovery. Please see our PLOS Data Policy page for detailed information on this policy.</p><br>A Data Availability	No datasets were generated or analyzed during the current study. De-identified data generated from the Delphi rounds and pilot testing will be made available upon study completion.

Statement, detailing where the data can be accessed, is required at first submission. Insert your Data Availability Statement in the box below.</p><br><br>Please see the data reporting of our submission guidelines for instructions on what you need to include in your Data Availability Statement. Examples of acceptable Data Availability Statements are included here for reference:</p><br> <br> - The minimal data set [and accompanying code, where generated] is available at [repository name] via [DOI hyperlink] <br> - No data was generated by this study. The following existing data sources were used: [dataset name] from [dataset location] available via [web address or DOI hyperlink]. <br> - For Study Protocols: No datasets were generated or analysed during the current study. All relevant data from this study will be made available upon study completion. <br> <br><br><i>PLOS allows rare exemptions to address legal and ethical concerns. If you have legal or ethical restrictions, please use the box below to detail these in full sentences for the Journal team to consider.</i></p><br><br>This statement will be typeset if the manuscript is accepted for publication.</p>	

Powered by Editorial Manager® and ProduXion Manager® from Aries Systems Corporation

Cover Letter

Click here to access/download;Manuscript;THESES-M Protocol PLoS ONE RG.docx

THESES-M: protocol for the development of a reporting guideline for medical theses

Carlos Rivero-López,[1] Ana Elena Limón-Rojas,[1] Ricardo Octavio Morales-Carmona,[1] Claudia Jimena Vilchis-Macedo,[1] Liz Hamui-Sutton,[1] Magali Fabiola Vega-Rodríguez,[2] Luisa Fernanda Romero-Henríquez,[3] Geovani López-Ortiz[2*]

[1] Division of Postgraduate Studies, Faculty of Medicine, National Autonomous University of Mexico, Mexico City 04510, Mexico.

[2] Department of Family Medicine, Faculty of Medicine, National Autonomous University of Mexico, Mexico City 04510, Mexico.

[3] Center for Teacher Professional Development in Mexico City, Ministry of Public Education, Mexico City 06400, Mexico.

* Corresponding author: geovani.lorz@fmposgrado.unam.mx

Funding

This research did not receive specific funding.

Competing interests

The authors declare that they have no competing interests.

Data availability

De-identified data derived from the Delphi rounds and pilot testing will be made publicly available upon study completion.

Abstract

Introduction: Medical theses represent one of the most widely produced yet least standardized forms of health sciences research. Their structure and reporting vary substantially across institutions, programs, and regions, and no formal reporting criteria exist to guide how these documents should be produced. This variability affects the consistency and evaluation of medical theses and may influence subsequent research reporting practices. This study describes the protocol for the development of THESES-M (Transparent and Holistic Evaluation Standards for Educational Studies in Medicine), a reporting guideline for medical theses.

Materials and methods: THESES-M will be developed in accordance with the guidance proposed by Moher et al. and the recommendations of the EQUATOR Network. The study will include a literature review to identify structural components of medical theses, generation of a preliminary list of reporting items, and three Delphi rounds with an expert panel using a 1–10 Likert scale. Consensus thresholds are predefined (≥80% rating ≥7 for inclusion; ≥80% rating ≤4 for exclusion). Agreement and stability will be assessed using median scores, interquartile ranges, and Kendall's coefficient of concordance. Items without consensus will be discussed in an in-person consensus meeting. The study outputs will include a consensus-based checklist and an explanation and elaboration document. Both will undergo pilot testing with medical students to assess clarity, feasibility, and usability.

Ethics and dissemination: Ethical approval was obtained from the Research and Ethics Committees of the Faculty of Medicine, National Autonomous University of Mexico (FM/DI/068/2025). Study findings will be disseminated through peer-reviewed publications and submission of the final guideline to the EQUATOR Network. THESES-M is registered with the EQUATOR Network as a reporting guideline under development.

Keywords: Medical Theses; Medical Education; Research Training; Reporting Guidelines; Academic Writing.

Introduction

Medical theses are widely used in health sciences education as formal research documents and, in many academic contexts, remain a requirement for degree completion. Their preparation involves the integration of methodological, argumentative, and scientific writing skills, and they often represent one of the earliest structured forms through which research is documented and communicated [1–4]. Despite their widespread use, medical theses are reported inconsistently across institutions, programs, and regional settings [5–7]. This heterogeneity affects the clarity, structure, and evaluability of these documents and has implications for how research is communicated in later academic and professional contexts.

The absence of standardized reporting criteria coexists with longstanding concerns regarding the quality, use, and dissemination of theses [8,9]. Without explicit guidance on what information should be reported and how it should be organized, medical theses may differ substantially in structure, content, and methodological detail, even when addressing the same study design. This variability weakens the consistency of the final document and may contribute to the low proportion of theses that are subsequently converted into scientific publications [9–11]. Deficiencies in the methodological reporting of medical theses have been documented across institutional settings, and thesis supervisors have identified structural and methodological problems as primary factors in the failure of thesis projects [6,9]. These findings suggest that the absence of explicit reporting criteria has measurable consequences for the consistency and evaluability of these documents.

In response to this gap, many institutions have developed internal guidance for thesis preparation. However, these documents are usually limited in scope and tend to focus on formal presentation requirements, such as cover-page format, citation style, or length, rather than on minimum standards for methodological reporting. This contrasts with the progress achieved in other areas of medical research, where reporting guidelines have been developed for multiple study designs, mainly through the EQUATOR Network [12]. These initiatives have established minimum standards for transparency,

reproducibility, and completeness in scientific reporting, thereby supporting critical appraisal and use in evidence synthesis [13–15]. These principles are also relevant to medical theses, where clear reporting of the study rationale, methods, results, and ethical considerations is essential for adequate academic evaluation and scientific communication [5,7]. Extending these standards to medical theses requires recognition that academic work produced during training also demands explicit and complete reporting.

Although the requirement to submit a thesis for degree completion varies across educational systems and regions, medical theses remain a formal requirement in a substantial number of countries across Europe, Latin America, Asia, and Africa, representing a significant volume of health sciences research produced at the formative stage of medical training [1,3,5,8,10]. Transparent and complete reporting matters in this context not only for academic evaluation but for the broader goal of communicating research with sufficient clarity to support replication and use.

Medical theses share these reporting needs, but they also include components that extend beyond the description of the study itself, such as the statement of the problem, the study rationale, and the regulatory and ethical considerations that support the work. In this context, the THESES-M initiative (Transparent and Holistic Evaluation Standards for Educational Studies in Medicine) proposes the development of a reporting guideline for medical theses. The initiative aims to establish a structured framework for defining the essential sections and content of these documents across different levels of medical training and methodological approaches.

Materials and Methods

Scope of the guideline

In this study, medical theses are defined as formally supervised academic research projects developed within undergraduate or postgraduate medical training programs and submitted as a requirement for the award of an academic degree. This definition includes theses addressing different thematic areas

(clinical, educational, health systems, or other medicine-related fields), regardless of methodological approach, as part of the research processes embedded in medical education.

Aim

To develop a reporting guideline for medical theses using a structured process that includes literature review, Delphi consensus rounds, a consensus meeting, and pilot testing.

Study design

This is a methodological study for the development of a reporting guideline, conducted in accordance with the guidance proposed by Moher et al. and the recommendations of the EQUATOR Network [16]. The process is organized into sequential phases with iterative components, including a literature review, Delphi rounds, a consensus meeting, and a process culminating in pilot testing. This approach is intended to identify, appraise, and refine the items required for the final guideline.

Setting

The study will be conducted at the Faculty of Medicine of the National Autonomous University of Mexico (UNAM). Consensus activities are expected to take place primarily in person at the institution's academic facilities.

EQUATOR registration

The THESES-M guideline is registered in the Reporting Guidelines Under Development section of the EQUATOR Network [17].

Development framework

THESES-M will be developed in accordance with the five phases described by Moher et al.:[16]

1. Initial steps: identification of need, definition of scope, and preliminary literature review.

2. Pre-meeting activities: generation of the preliminary list of items for checklist development, selection of experts, distribution of materials, and completion of three Delphi rounds.
3. Consensus meeting: structured discussion and in-person refinement of the items.
4. Post-meeting activities: consolidation of agreements and pilot testing.
5. Post-publication considerations: strategies for dissemination, institutional adoption, and future updating.

Phase 1: Initial steps

Identification of need and scope

THESES-M is intended to address the absence of reporting standards specifically designed for medical theses. It seeks to define the minimum reporting criteria applicable to degree-granting theses in medicine, regardless of level of training or methodological approach.

Literature review

A literature review using systematic procedures will be undertaken to identify recurring components in the reporting of medical theses. The search will be carried out independently by CRL and GLO in PubMed, ERIC, Google Scholar, and institutional repositories of medical schools, using combinations of Spanish- and English-language terms related to "medical thesis," "dissertation guidelines," "medical education," "reporting," and "structure." Search strategies, dates, and the number of records retrieved will be documented.

Institutional guidelines and policy documents explicitly describing the structure or evaluation criteria for medical theses, as well as relevant methodological literature, will be included. Selection will be performed independently by two reviewers (CRL and GLO) in two sequential stages: first, screening of titles, abstracts, or institutional descriptions to assess initial relevance; second, full-text review for inclusion. Disagreements will be resolved by consensus and, if necessary, by consultation with a third reviewer (LFRH). To reduce bias related to institutional availability, the open search will be

supplemented by a targeted search by geographic region and training program type, incorporating public and private institutions whenever information is available. The number of documents identified, excluded, and finally included will be recorded, along with the country or region of the corresponding institutions.

In the second stage, structural and content elements will be extracted independently using a standardized matrix (institution, level of training, document type and year, required sections, methodological requirements, and ethical standards). Discrepancies will be resolved through adjudication. Initial agreement between reviewers will be documented for each selection stage. The kappa coefficient will be calculated when both reviewers have independently assessed the same set of full-text documents, provided that the distribution of decisions allows for a meaningful estimate. The extracted elements will be synthesized in a thematic matrix to generate the preliminary list of items, with explicit traceability to their sources. No formal quality appraisal will be undertaken; the analysis will focus on identifying and comparing the structural components and stated criteria in the documents included.

Generation of preliminary items

Based on the literature review, the research team will generate an initial list of items through the following process:

1. Extraction: three researchers will independently extract potential elements from the reviewed documents.
2. Consolidation: the extracted elements will be consolidated by removing duplicates and grouping similar concepts.
3. Categorization: the items will be organized according to the typical sections of a thesis (e.g., introduction, problem statement and research question, methods, ethical considerations, results, discussion).

4. Initial consensus: the full team will review the consolidated list through iterative meetings until consensus is reached on the version to be submitted to the Delphi panel. This preliminary version will include operational definitions for each item and will serve as the initial input for the Delphi rounds.

The result of this process will constitute the preliminary version of the THESES-M checklist, which will be used as the initial input for the Delphi rounds.

Phase 2: Pre-meeting activities

Panel selection

Purposive sampling will be used to recruit experts from the following key groups:

1. Specialists in medical education
2. Researchers
3. Methodologists
4. Thesis supervisors (undergraduate and postgraduate)

Eligibility criteria

Inclusion criteria:

a. A minimum of 5 years of experience in the supervision or evaluation of medical theses.
b. Participation in formal academic evaluation or research processes.
c. Familiarity with scientific reporting guidelines and thesis development processes.

Exclusion criteria:

a. Lack of informed consent to participate.
b. Inability to participate in the Delphi process.
c. Absence of verifiable experience in thesis supervision, evaluation, or methodological review.

This composition will enable the integration of complementary perspectives relevant to the development of the guideline. Recruitment will seek geographic diversity (across different regions of the country and, where feasible, other countries), institutional diversity (public and private universities), and methodological diversity (experts with experience in evaluating clinical, educational, and health systems studies). The process will begin with direct invitations to identified experts and will be complemented by participant nominations to identify colleagues whose profiles are not yet represented. The final composition of the panel will be documented.

Participants

Approximately 30 experts will be recruited (target range: 28–32). This range is consistent with the guidance and practice described by Moher et al., whose framework for reporting guideline development has employed iterative Delphi processes with panels ranging from small expert groups to over 100 participants depending on the scope and representativeness required [16]. The target range for THESES-M reflects a balance between ensuring representation across the four predefined key groups and maintaining operational feasibility across three Delphi rounds. Response rates for each round and, where applicable, the attrition rate will be reported, together with the documented reasons.

Delphi rounds

Three Delphi rounds will be conducted to assess the importance of proposed items and to establish consensus on their inclusion, exclusion, or revision [18].

Delphi Round 1

Panelists will receive the preliminary set of items, their operational definitions, instructions, and evaluation criteria. Using a 1-to-10 Likert scale (1 = lowest importance, 10 = highest importance), they will independently rate each item's importance. The assessment will be conducted through an electronic form (Google Forms). Spaces will be provided for qualitative comments and suggestions on modifications or the inclusion of new items.

Importance categories

For operational purposes, Likert scores will be grouped into four importance categories, following methodological precedents used in consensus studies [19-21]. This classification will be used solely to facilitate the interpretation of the scores during the Delphi rounds.

- 1–4: low importance (the item should not be included)
- 5–6: moderate importance (its inclusion could be considered)
- 7–8: high importance (likely to be included)
- 9–10: very high importance (essential for inclusion)

Consensus criteria

An item will be considered to have reached consensus when any of the following criteria are met:

- Consensus for inclusion: ≥80% of panelists rate the item ≥7, combining the categories of high importance (7–8) and very high importance (9–10).
- Consensus for exclusion: ≥80% of panelists rate the item ≤4, corresponding to the category of low importance.
- No consensus: neither of the above criteria is met.

Item handling according to consensus status

- Items reaching consensus for inclusion (≥80% rated ≥7): will be included in the preliminary version of the checklist and will not be re-evaluated in subsequent rounds.
- Items reaching consensus for exclusion (≥80% rated ≤4): will be proposed for definitive exclusion.
- Items without consensus: will be retained for re-evaluation in subsequent rounds. If disagreement persists after the third round, they will be discussed and resolved by structured voting during the consensus meeting.

Additional measures

The median and interquartile range (IQR) of the scores will be calculated for each item. This will allow quantification of the degree of dispersion in the ratings and provide structured feedback to panelists in subsequent rounds.

Items may be reworded between rounds if qualitative comments consistently indicate ambiguity or lack of clarity. All modifications will be documented in the feedback report for the subsequent round, specifying the original version, the rationale for the change, and the revised version. Panelists may suggest new items; those proposed independently by at least three panelists from different groups will be included for evaluation in the next round.

Qualitative comments from each round will be analyzed through thematic coding by two investigators independently, identifying patterns of ambiguity, suggestions for rewording, or proposals for new items. Discrepancies will be resolved by consensus.

In addition, consensus stability across rounds will be assessed by calculating Kendall's coefficient of concordance (W) for items evaluated in multiple rounds, with W values ≥ 0.8 considered indicative of robust consensus. The percentage of items whose consensus classification changes between consecutive rounds will be documented as an indicator of the deliberative process's evolution.

Delphi Round 2

Items that do not reach consensus in the first round, or that are modified on the basis of qualitative comments, will be resubmitted for a second evaluation. Panelists will receive an anonymized summary including the distribution of scores from the previous round (median, interquartile range, and frequencies), a synthesis of qualitative comments grouped thematically, and their own previous rating. The same 1-to-10 scale, importance categories, and consensus criteria defined for the first round will be used. Items that remain without consensus will proceed to the third Delphi round.

Delphi Round 3

In the third round, items that have not reached consensus in the previous rounds will be re-evaluated. Panelists will receive an updated summary of the scores and comments accumulated across the first two rounds. The same scale, categories, and consensus criteria will be maintained. Items that remain without consensus will be reserved for resolution by structured voting during the in-person or synchronous virtual consensus meeting.

Phase 3: Consensus meeting

Consensus activities will be conducted in person at the Faculty of Medicine of the National Autonomous University of Mexico, with synchronous remote participation enabled if required for panelists unable to attend on-site. During the meeting, the results of previous activities will be presented, including summaries of the Delphi rounds and the relevant evidence used to develop the items.

The activities will include:

- Presentation of the results of the Delphi rounds.
- Analysis of the methodological justification for the included items.
- Review of the proposed order and the thematic relationship between sections.
- Definition of a flow diagram for presentation of the checklist.
- Agreements on responsibilities for drafting the explanation and elaboration document.

The outcome of the meeting will be a consolidated version of the checklist and a detailed work plan for the development of the explanation and elaboration document and dissemination materials.

Outcomes

The primary outcome of this study is the THESES-M checklist: a consensus-based set of reporting items for medical theses, defined as those items for which ≥80% of panelists assign a score of ≥7 on

a 1-to-10 Likert scale across the Delphi rounds and confirmed during the consensus meeting. The checklist will be produced at the end of Phase 3 and consolidated during Phase 4.

The secondary outcome is the explanation and elaboration document, which constitutes the instrument supporting the checklist. For each item, it will provide a definition, a rationale for its inclusion, and annotated examples to guide its interpretation and application. Its clarity, comprehensibility, and feasibility will be evaluated during pilot testing (Phase 4) through a structured questionnaire administered to participating students.

Phase 4: Post-meeting activities

Following the consensus meeting, the team will integrate the agreements reached to produce the checklist version derived from the consensus process. This version will incorporate the items accepted during the Delphi rounds and the editorial adjustments defined during the meeting. Its purpose will be to structure the document that will be evaluated through pilot testing.

Development of the explanation and elaboration document

The team will prepare the explanation and elaboration document, incorporating definitions, clarifications, and examples to facilitate the interpretation of each item. Consistency between the checklist and the content of the explanation and elaboration document will be reviewed to ensure conceptual precision and internal coherence.

Pilot testing

The checklist and the explanation and elaboration document resulting from the consensus process will undergo pilot testing with the following aims: (a) to assess the clarity of the items and their operational definitions; (b) to verify that the explanations make it possible to understand what information should be reported to satisfy each item; (c) to estimate the time required to complete the checklist; (d) to identify feasibility barriers in its application; and (e) to explore its perceived

usefulness and acceptability as a reporting tool. This preliminary evaluation will provide information on the instrument's usability in real-world conditions.

Approximately 20–25 students will participate in pilot testing. Inclusion criteria will comprise students currently enrolled in undergraduate, residency, master’s, or doctoral medical training who have completed data collection and analysis and are in the thesis-writing stage. Each participant will receive the checklist together with the explanation and elaboration document and will apply both to their own thesis. For each item, they will assess: (a) whether the wording is clear; (b) whether the explanation provided allows them to identify what information should be reported; (c) whether it is feasible to apply it to their type of study; and (d) suggestions for improvement. In addition, they will complete a structured questionnaire including time spent applying the tool, difficulties encountered, perceived usefulness and acceptability of the explanation and elaboration document, and practical barriers to implementation.

The observations will be systematized through descriptive and thematic content analyses to identify recurring patterns of ambiguity or comprehension problems.

Data management plan

Data generated during the Delphi rounds and pilot testing will be stored in de-identified form in password-protected institutional files accessible only to the research team. Any information required for participant contact or follow-up will be stored separately from response data. Only aggregated results will be reported, and access to study data will be subject to ethical and confidentiality restrictions.

Safety considerations

This study does not involve clinical interventions, biological samples, or procedures that may pose risks to participants. Safety considerations are therefore not applicable.

Implementation materials

Templates and support resources will be prepared to facilitate institutional adoption of THESES-M, including brief guides and instructions for use by students and thesis supervisors.

Final consolidation

The team will integrate the agreements reached during the consensus process and the feedback obtained during pilot testing to produce the final version of the checklist and the explanation and elaboration document. The THESES-M checklist and its explanation and elaboration document will be submitted for inclusion in the EQUATOR Network and published with open access to support international dissemination.

Ethics

Ethical approval was obtained from the Research Committee and the Research Ethics Committee of the Faculty of Medicine of the National Autonomous University of Mexico (FM/DI/068/2025). Participation in the Delphi rounds and pilot testing will be voluntary, and all participants will provide written informed consent prior to their inclusion. Data will be treated in accordance with confidentiality standards; responses will be anonymized during analysis and only aggregated results will be reported.

Study status and timeline

At the time of manuscript submission, the study is in the protocol phase. The literature review and preliminary item generation (Phase 1) will be conducted between 1 June 2026 and 31 July 2026. Experts for the Delphi panel will be prospectively recruited between 1 September 2026 and 30 November 2026, during which the three Delphi rounds (Phase 2) will be conducted. The consensus meeting (Phase 3) is scheduled between 1 February 2027 and 28 February 2027. Participants for pilot testing will be prospectively recruited between 1 March 2027 and 30 June 2027 (Phase 4). All

participants, including Delphi panel experts and students involved in pilot testing, will provide written informed consent, obtained electronically through an online form prior to participation. Participants will only gain access to the study materials after providing consent. Participation will be voluntary and data will be collected anonymously. No minors will be included in this study. Final consolidation and dissemination activities (Phase 5) will take place between 1 July 2027 and 31 August 2027.

Phase 5: Post-publication activities

Following publication of THESES-M, actions will be established to ensure accessibility, appropriate use, and ongoing updating of the guideline. A dedicated digital repository will be set up to host the checklist, the explanation and elaboration document, implementation materials, and version records. Its purpose will be to provide a single point of access and facilitate open availability.

The team will promote its availability to academic programs and units responsible for research training to facilitate its integration into thesis supervision, evaluation, and reporting processes. Support materials—such as templates and annotated examples—will also be developed to facilitate interpretation of the checklist across different levels of training.

Official translations will also be developed through a translation/back-translation process and will be published in the repository with their corresponding version records. The repository will include a mechanism for receiving user feedback, enabling the identification of interpretive difficulties and areas requiring adjustment. In parallel, the first experiences of institutional implementation will be documented to identify patterns of use and potential critical issues.

Finally, a process of periodic review will be established to incorporate new evidence, clarify ambiguous elements, and update the content to address identified needs. All modifications will be recorded in the repository to ensure traceability and transparency.

Discussion

THESES-M seeks to address a documented gap in the academic infrastructure for reporting medical theses [7]. Although reporting guidelines have transformed scientific communication across multiple fields [15,22,23], no equivalent standards currently exist for medical theses as academic documents. By establishing explicit reporting criteria, this project aims to provide a structured framework to support greater consistency in the evaluation and reporting of medical theses.

The proposed methodology combines literature review, Delphi consensus rounds, a consensus meeting, and pilot testing to support the iterative development of the guideline [16–19]. This process is intended to refine the proposed items through the integration of complementary perspectives from medical educators, researchers, methodologists, and thesis supervisors, thereby strengthening the conceptual clarity, relevance, and usability of the final checklist and its explanation and elaboration document.

THESES-M may contribute to more explicit reporting practices in medical education from early formative stages. A transparent and clearly defined reporting structure does more than organize information; it also clarifies the study's internal logic and situates the student’s work within recognized scientific conventions [5,7]. A structured reporting framework may facilitate clearer academic evaluation and more consistent communication of these documents. At the same time, adherence to a reporting guideline does not in itself guarantee methodological quality; rather, it supports more transparent and evaluable reporting. Its usefulness will depend on factors such as institutional adoption, user adherence, and the characteristics of the training environments in which it is implemented.

Transparent reporting is also relevant from an ethical perspective. Its absence limits critical appraisal, weakens reproducibility, and reduces the usability of academic work [24–26]. By defining minimum standards of clarity, completeness, and explicit reporting, THESES-M aligns with principles

recognized by the EQUATOR Network and extends their application to the field of research training in medicine [15,23,26].

The implementation of a reporting guideline for medical theses may also encounter institutional and cultural resistance. Standards may be perceived as limiting methodological flexibility or as introducing forms of homogenization that do not fully account for disciplinary variation or differences across training contexts. Institutions with established traditions in thesis supervision and evaluation may also regard external guidance as interference with existing academic practices. These concerns require explicit recognition, because the value of a reporting guideline lies in making research communication more explicit and comparable, not in prescribing a single methodological model. In this sense, THESES-M is intended to operate at the level of reporting rather than at the level of methodological choice.

This study has limitations that should be acknowledged. The composition of the expert panel may influence the prioritization of items included in the final guideline, despite efforts to ensure disciplinary, methodological, institutional, and geographic diversity. In addition, the identification of institutional guidance documents may depend on their public availability, which could restrict the range of materials included in the literature review. Pilot testing will be conducted with students who are already in the thesis-writing stage, so the evaluation will primarily reflect usability at that phase rather than across the entire research process.

Any substantial modifications to the study procedures after protocol publication will be documented and reported in subsequent outputs arising from the study.

Overall, THESES-M seeks to establish minimum standards for the reporting of medical theses. By specifying the essential sections and content that should be included in these documents, the guideline is intended to provide a structured basis for clearer and more explicit academic and scientific

communication. Its implementation, institutional uptake, and effects on reporting practices will need to be evaluated in subsequent studies.

Authors' contributions

CRL, LFRH, and GLO: Conceptualization, Methodology, Writing – original draft. AELR, ROMC, CJVM, LHS, and MFVR: Methodology, Writing – review & editing. All authors read and approved the final version of the manuscript.

Acknowledgements

Not applicable.

References

1. Nieminen P, Sipilä K, Takkinen HM, Renko M, Risteli L. Medical theses as part of the scientific training in basic medical and dental education: experiences from Finland. BMC Med Educ. 2007;7(1):51. doi:10.1186/1472-6920-7-51.

2. Fabre A. Publication des thèses d'exercice de pédiatrie. Arch Pediatr. 2015;22(8):802–6. doi:10.1016/j.arcped.2015.03.013.

3. Sahoo AK, Mishra N, Jain M, Sahoo M, Ghoshal P. Problems and solutions to conduct of thesis of postgraduate medical students during the COVID-19 pandemic: an insight into the students perspective. Korean J Med Educ. 2023;35(1):55–70. doi:10.3946/kjme.2023.249.

4. Mahajan R. Confusion Surrounding the Mandatory Requirement of Thesis Submission: Review of the Postgraduate Medical Education Regulations of the National Medical Commission. Int J Appl Basic Med Res. 2025;15(1):1–3. doi:10.4103/ijabmr.ijabmr_558_24.

5. Tokatlı M, Balcı C. Academic outcomes of geriatric medicine theses in internal medicine residency: a 25-year nationwide review in Türkiye. BMC Med Educ. 2025;26(1):100. doi:10.1186/s12909-025-08442-7.

6. Ullrich C, Stürmlinger A, Wensing M, Krug K. Qualitative research methods in medical dissertations: an observational methodological study on prevalence and reporting quality of dissertation abstracts in a German university. BMC Med Res Methodol. 2020;20(1):301. doi:10.1186/s12874-020-01186-6.

7. Griegel S, Kühl M, Schneider A, Kühl SJ. Medical dissertation basics: analysis of a course of study for medical students. GMS J Med Educ. 2022;39(2):Doc26. doi:10.3205/ZMA001547.

8. Yahia F, Gazzeh H, Ben Mansour F, Zanina Y, Neffati E, Ben Abdelaziz A. From Thesis to Publication: Unveiling the Predictive Factors for Cardiology Research at a North African Faculty of Medicine (Tunisia). Tunis Med. 2024;102(11):858-865. doi:10.62438/tunismed.v102i11.5230.

9. Can E, Richter F, Valchanova R, Dewey M. Supervisors' perspective on medical thesis projects and dropout rates: survey among thesis supervisors at a large German university hospital. BMJ Open. 2016;6(10):e012726. doi:10.1136/bmjopen-2016-012726.

10. Touissi Y, Boulaich O, El Idrissi FE, Hjiej G, Stitou O, Belakbyer H, et al. Medical students' contribution to research; the scientific output of medical theses held in Moroccan medical schools during the last decade (2011-2021). Med Educ Online. 2023;28(1):2218677. doi:10.1080/10872981.2023.2218677.

11. Björklund M, Massoumi R, Ohlsson B. From master's thesis to research publication: a mixed-methods study of medical student publishing and experiences with the publishing process. BMC Med Educ. 2024;24(1):75. doi:10.1186/s12909-024-05060-7.

12. EQUATOR Network. Enhancing the QUAlity and Transparency Of Health Research [Internet]. [cited 2026 Jan 31]. Available from: https://www.equator-network.org/

13. Von Elm E, Altman DG, Egger M, Pocock SJ, Gøtzsche PC, Vandenbroucke JP. The Strengthening the Reporting of Observational Studies in Epidemiology (STROBE) statement: guidelines for reporting observational studies. J Clin Epidemiol. 2008;61(4):344–9. doi:10.1016/j.jclinepi.2007.11.008.

14. Hopewell S, Chan AW, Collins GS, Hróbjartsson A, Moher D, Schulz KF, et al. CONSORT 2025 statement: updated guideline for reporting randomised trials. BMJ. 2025;389:e081123. doi:10.1136/bmj-2024-081123.

15. Fragoso-Mendoza MI, Dávila-Mendoza R, López-Ortiz G. Importance and use of guidelines to report main types of studies in medical research. Cir Cir. 2023;91(2):277-283. doi:10.24875/CIRUE.M22000518.

16. Moher D, Schulz KF, Simera I, Altman DG. Guidance for Developers of Health Research Reporting Guidelines. PLoS Med. 2010;7(2):e1000217. doi:10.1371/journal.pmed.1000217.

17. EQUATOR Network. Guidelines Under Development. [Internet]. [cited 2026 Jan 31]. Available from: https://www.equator-network.org/library/reporting-guidelines-under-development/reporting-guidelines-under-development-for-other-study-designs/#THESES-M.

18. Murphy MK, Black NA, Lamping DL, McKee CM, Sanderson CF, Askham J, et al. Consensus development methods, and their use in clinical guideline development. Health Technol Assess. 1998;2(3):i–iv, 1–88. PMID: 9561895.

19. Portney LG, Watkins MP. Foundations of clinical research: applications to practice. 3rd ed. Upper Saddle River (NJ): Pearson/Prentice Hall; 2009.

20. Phillips AC, Lewis LK, McEvoy MP, Galipeau J, Glasziou P, Hammick M, et al. Protocol for development of the guideline for reporting evidence based practice educational interventions and teaching (GREET) statement. BMC Med Educ. 2013;13(1):9. doi:10.1186/1472-6920-13-9.

21. Phillips AC, Lewis LK, McEvoy MP, Galipeau J, Glasziou P, Moher D, Tilson JK, Williams MT. Development and validation of the guideline for reporting evidence-based practice educational interventions and teaching (GREET). BMC Med Educ. 2016;16(1):237. doi: 10.1186/s12909-016-0759-1.

22. Rulli D. The Role of Reporting Guidelines in Research Publication. J Dent Hyg. 2025;99(1):63–7. PMID: 39929523.

23. Altman DG, Simera I. A history of the evolution of guidelines for reporting medical research: the long road to the EQUATOR Network. J R Soc Med. 2016;109(2):67–77. doi:10.1177/0141076815625599.

24. Ahmady S, Kohan N, Hamidi H, Vahednasiri A, Hooshmandja M. Interpretations of reproducibility crisis in medical education research: a qualitative study. Sci Rep. 2026;16(1):4489. doi:10.1038/s41598-025-34640-w.

25. Haven TL, Ioannidis JPA. Reproducibility Failure in Biomedical Research: Problems and Solutions. Annu Rev Med. 2026;77(1):537–49. doi:10.1146/annurev-med-050124-050859.

26. Simera I, Moher D, Hirst A, Hoey J, Schulz KF, Altman DG. Transparent and accurate reporting increases reliability, utility, and impact of your research: reporting guidelines and the EQUATOR Network. BMC Med. 2010;8(1):24. doi:10.1186/1741-7015-8-24.
