# Supplementary material for: THESES-M: protocol for the development of a reporting guideline for medical theses
Source: PLoS One. 2026 Aug 7;21(8):e0354899. doi: 10.1371/journal.pone.0354899 (PMC13450800; doi:10.1371/journal.pone.0354899)
Supplement: Supplementary file 3 [file pone.0354899.s002.docx]

**Title: THESES-M: protocol for the development of a reporting guideline for medical theses**

Rationale: The manuscript explains the gap (the absence of uniform reporting standards for medical theses) and explains why THESES-M is necessary. The reasoning is backed by relevant literature that shows variations in thesis structure, low publication conversion rates, and faults noted by supervisors. The goal is clearly stated: to create a reporting guideline utilizing proven EQUATOR Network techniques.

Although the protocol is generally sound, there are a few areas that need to be strengthened or clarified before I would say it is completely robust:
The majority of methodological literature uses >0.5–0.7, although the Delphi consensus level for stability (Kendall's W ≥ 0.8) is exceptionally strict. This needs to be clarified or updated.
It is unclear how to handle "moderate importance" elements (scoring 5–6); clear guidelines are required.
For the three rounds of the Delphi panel, no attrition management strategy is outlined.
The qualitative analysis of comments (e.g., coding scheme, inter-rater reliability targets) is stated but not explained.
These problems should be fixed to avoid unreported flexibility, but they do not render the protocol illegal.

Methodology: The technique is sufficiently detailed for replication and adheres to the Moher et al. framework:
The literature review search strategy is devoid of precise search terms, time constraints, and a methodical approach to locating institutional repositories. Replicability is so limited.
There is insufficient information about the pilot testing questionnaire (e.g., scales, validation).
There is no discussion of how to handle institutional guidelines that are not in English.
The Delphi panel's sample size rationale (28–32 experts) is descriptive rather than empirically supported. Replicability would be significantly enhanced by fixing these omissions.

The work employs standard scientific English and is arranged and written clearly. Throughout, the language is understandable.

As a Reviewer, i believe Sample size justification for the Delphi panel needs strengthening. Literature review methodology lacks search strings and a systematic approach to institutional repositories. Also, consensus thresholds (Kendall’s W ≥ 0.8) are too stringent; handling of scores 5–6 is ambiguous. Pilot testing design not mentioned appropriately.

Conflict of interest/disclosure is missing, qualitative analysis plan, patient/public involvement rationale.

Reference style is truncated (page 27).

Data availability statement does not comply with PLOS ONE policy (needs repository and timing clarification).

I recommend minor revision.

Reviewer 2
