# Supplementary material for: THESES-M: protocol for the development of a reporting guideline for medical theses
Source: PLoS One. 2026 Aug 7;21(8):e0354899. doi: 10.1371/journal.pone.0354899 (PMC13450800; doi:10.1371/journal.pone.0354899)
Supplement: Peer review history

# Peer review: Decision Letter 0

**DOI:** 10.1371/journal.pone.0354899.r001

**Authors:** Paolo Landa

20 May 2026

PONE-D-26-15953

THESES-M: protocol for the development of a reporting guideline for medical theses

-->PLOS One

Dear Dr. López-Ortiz,

Thank you for submitting your manuscript to PLOS ONE. After careful consideration, we feel that it has merit but does not fully meet PLOS ONE’s publication criteria as it currently stands. Therefore, we invite you to submit a revised version of the manuscript that addresses the points raised during the review process.

ACADEMIC EDITOR:  The reviewers asked minor revisions of the manuscript

Please submit your revised manuscript by Jul 04 2026 11:59PM. If you will need more time than this to complete your revisions, please reply to this message or contact the journal office at plosone@plos.org. When you're ready to submit your revision, log on to https://www.editorialmanager.com/pone/ and select the 'Submissions Needing Revision' folder to locate your manuscript file.

Please include the following items when submitting your revised manuscript:

- A letter that responds to each point raised by the academic editor and reviewer(s). You should upload this letter as a separate file labeled 'Response to Reviewers'.
- A marked-up copy of your manuscript that highlights changes made to the original version. You should upload this as a separate file labeled 'Revised Manuscript with Track Changes'.
- An unmarked version of your revised paper without tracked changes. You should upload this as a separate file labeled 'Manuscript'.

If you would like to make changes to your financial disclosure, please include your updated statement in your cover letter. Guidelines for resubmitting your figure files are available below the reviewer comments at the end of this letter.

If applicable, we recommend that you deposit your laboratory protocols in protocols.io to enhance the reproducibility of your results. Protocols.io assigns your protocol its own identifier (DOI) so that it can be cited independently in the future. For instructions see: https://journals.plos.org/plosone/s/submission-guidelines#loc-laboratory-protocols. Additionally, PLOS ONE offers an option for publishing peer-reviewed Lab Protocol articles, which describe protocols hosted on protocols.io. Read more information on sharing protocols at https://plos.org/protocols?utm_medium=editorial-email&utm_source=authorletters&utm_campaign=protocols.

As the corresponding author, your ORCID iD is verified in the submission system and will appear in the published article. PLOS supports the use of ORCID, and we encourage all coauthors to register for an ORCID iD and use it as well. Please encourage your coauthors to verify their ORCID iD within the submission system before final acceptance, as unverified ORCID iDs will not appear in the published article. *Only*  the individual author can complete the verification step; PLOS staff *cannot*  verify ORCID iDs on behalf of authors.

We look forward to receiving your revised manuscript.

Kind regards,

Paolo Landa, Ph.D.

Academic Editor

PLOS One

Journal Requirements:

When submitting your revision, we need you to address these additional requirements.

1. Please ensure that your manuscript meets PLOS ONE's style requirements, including those for file naming. The PLOS ONE style templates can be found at

https://journals.plos.org/plosone/s/file?id=wjVg/PLOSOne_formatting_sample_main_body.pdf and

https://journals.plos.org/plosone/s/file?id=ba62/PLOSOne_formatting_sample_title_authors_affiliations.pdf

2. When completing the data availability statement of the submission form, you indicated that you will make your data available on acceptance. We strongly recommend all authors decide on a data sharing plan before acceptance, as the process can be lengthy and hold up publication timelines. Please note that, though access restrictions are acceptable now, your entire data will need to be made freely accessible if your manuscript is accepted for publication. This policy applies to all data except where public deposition would breach compliance with the protocol approved by your research ethics board. If you are unable to adhere to our open data policy, please kindly revise your statement to explain your reasoning and we will seek the editor's input on an exemption. Please be assured that, once you have provided your new statement, the assessment of your exemption will not hold up the peer review process.

3. If the reviewer comments include a recommendation to cite specific previously published works, please review and evaluate these publications to determine whether they are relevant and should be cited. There is no requirement to cite these works unless the editor has indicated otherwise.

4. Please review your reference list to ensure that it is complete and correct. If you have cited papers that have been retracted, please include the rationale for doing so in the manuscript text, or remove these references and replace them with relevant current references. Any changes to the reference list should be mentioned in the rebuttal letter that accompanies your revised manuscript. If you need to cite a retracted article, indicate the article’s retracted status in the References list and also include a citation and full reference for the retraction notice.

[Note: HTML markup is below. Please do not edit.]

Reviewers' comments:

Reviewer's Responses to Questions

**Comments to the Author**

1. Does the manuscript provide a valid rationale for the proposed study, with clearly identified and justified research questions?

The research question outlined is expected to address a valid academic problem or topic and contribute to the base of knowledge in the field.

Reviewer #1: Yes

Reviewer #2: Yes

Reviewer #3: Yes

Reviewer #4: Partly

Reviewer #5: Yes

**********

2. Is the protocol technically sound and planned in a manner that will lead to a meaningful outcome and allow testing the stated hypotheses?

The manuscript should describe the methods in sufficient detail to prevent undisclosed flexibility in the experimental procedure or analysis pipeline, including sufficient outcome-neutral conditions (e.g. necessary controls, absence of floor or ceiling effects) to test the proposed hypotheses and a statistical power analysis where applicable. As there may be aspects of the methodology and analysis which can only be refined once the work is undertaken, authors should outline potential assumptions and explicitly describe what aspects of the proposed analyses, if any, are exploratory.

Reviewer #1: Yes

Reviewer #2: Partly

Reviewer #3: No

Reviewer #4: Partly

Reviewer #5: Yes

**********

3. Is the methodology feasible and described in sufficient detail to allow the work to be replicable?

Descriptions of methods and materials in the protocol should be reported in sufficient detail for another researcher to reproduce all experiments and analyses. The protocol should describe the appropriate controls, sample size calculations, and replication needed to ensure that the data are robust and reproducible.

Reviewer #1: Yes

Reviewer #2: Yes

Reviewer #3: No

Reviewer #4: Yes

Reviewer #5: Yes

**********

4. Have the authors described where all data underlying the findings will be made available when the study is complete?

The PLOS Data policy requires authors to make all data underlying the findings described in their manuscript fully available without restriction, with rare exception, at the time of publication. The data should be provided as part of the manuscript or its supporting information, or deposited to a public repository. For example, in addition to summary statistics, the data points behind means, medians and variance measures should be available. If there are restrictions on publicly sharing data—e.g. participant privacy or use of data from a third party—those must be specified.

Reviewer #1: No

Reviewer #2: No

Reviewer #3: No

Reviewer #4: Yes

Reviewer #5: No

**********

5. Is the manuscript presented in an intelligible fashion and written in standard English?

PLOS ONE does not copyedit accepted manuscripts, so the language in submitted articles must be clear, correct, and unambiguous. Any typographical or grammatical errors should be corrected at revision, so please note any specific errors here.

Reviewer #1: Yes

Reviewer #2: Yes

Reviewer #3: Yes

Reviewer #4: Yes

Reviewer #5: Yes

**********

6. Review Comments to the Author

Please use the space provided to explain your answers to the questions above and, if applicable, provide comments about issues authors must address before this protocol can be accepted for publication. You may also include additional comments for the author, including concerns about research or publication ethics.

You may also provide optional suggestions and comments to authors that they might find helpful in planning their study.

(Please upload your review as an attachment if it exceeds 20,000 characters)

Reviewer #1: This manuscript presents a structured and methodologically sound protocol for developing the THESES-M reporting guideline, addressing the important gap of standardized reporting criteria in medical theses. The rationale is well-supported by relevant literature.

The methodology adheres to EQUATOR Network recommendations and includes a systematic literature review, Delphi rounds, predefined consensus criteria, and a structured consensus meeting, enhancing its robustness. Pilot testing ensures the checklist's feasibility and usability.

The methodology is detailed and reproducible, with transparent processes for literature review, item generation, expert selection, Delphi procedures, and data analysis. The combination of quantitative measures (median, IQR, Kendall’s W) and qualitative thematic analysis strengthens the consensus validity.

However, some areas need clarification:

1. Data Availability: Specify the repository for de-identified data to enhance transparency.

2. Literature Review Scope: Include more detail on inclusion/exclusion criteria and quality appraisal.

3. Expert Panel Diversity: Clarify how international representation beyond Mexico will be achieved.

4. Potential Bias: Discuss strategies to mitigate selection bias from relying on publicly available documents.

5. Minor Language Edits: Polishing some sections could improve readability.

Overall, this is a high-quality protocol following established standards and has potential contributions to medical education and research reporting. With minor revisions, it would be suitable for publication.

Reviewer #2: As a Reviewer, i believe Sample size justification for the Delphi panel needs strengthening. Literature review methodology lacks search strings and a systematic approach to institutional repositories. Also consensus thresholds (Kendall’s W ≥ 0.8) are too stringent; handling of scores 5–6 is ambiguous. Pilot testing design not mentioned appropriately.

Conflict of interest/disclosure is missing, qualitative analysis plan, patient/public involvement rationale.

Reference style is truncated (page 27).

Data availability statement does not comply with PLOS ONE policy (needs repository and timing clarification).

I recommend minor revision.

Reviewer #3: 1. Delphi Method - the protocol sets a threshold of more than 80% consensus. my recommendation is please define a clear "gray zone" management strategy. If an item receives 70–79% agreement, will it be automatically discarded, or will it be discussed in a virtual meeting? Defining the process for near-consensus items will prevent the loss of potentially valuable reporting standards.

2. Please specify the metrics for "feasibility." Will this be measured by the time it takes to complete the checklist, or by inter-rater reliability scores when two different evaluators use THESES-M to grade the same thesis? Strengthening the description of the pilot phase will provide more confidence in the guideline’s eventual validation.

3. LR - Please Include a "Knowledge Translation" or "Dissemination" section in the protocol. This could involve plans to develop an Explanation and Elaboration (E&E) document (similar to CONSORT or PRISMA) or strategies to partner with specific medical university networks to mandate the guideline’s use for graduation.

4. Please define a clear "gray zone" management strategy. If an item receives 70–79% agreement, will it be automatically discarded, or will it be discussed in a virtual meeting? Defining the process for near-consensus items will prevent the loss of potentially valuable reporting standards.

5. Please define an IQR ≤ 2 as the objective cutoff for consensus.

6. please measured dispersion (IQR) - Low IQR - please indicates the most experts ranting are clustered closed to gather (High consensus), High IQR - Please indicates that experts opinions are widely spread in low consensus.

7. Please add "change in IQR' alongside kendalls W to confirm when the the panel's opinion has stabilized

8. Please adding the percentage change in the IQR between rounds. If the IQR does not change significantly between Round 2 and Round 3, it proves "stability," meaning further rounds would not change the outcome. This adds a second layer of statistical rigor to your Delphi process.

9. please polishing the novelist

Reviewer #4: The manuscript is a study protocol describing the development of a guideline framework, THESES-M, with the intent to provide minimal standard requirements on consistency and evaluation of medical theses. The authors had identified this gap through the variability of how medical theses are evaluated and, especially, on the observation around the variability in how medical theses are structured, evaluated, and ultimately translated into scientific publications. The authors make the case that inconsistency in thesis quality may contribute to the relatively low rate of conversion into manuscripts, and their effort to develop a standardized framework is both timely and relevant. The strength of the work lies in its clear identification of this gap, as well as in the overall structured and replicable protocol presented. The inclusion of pilot testing with students is particularly valuable, as it grounds the framework in practical application and usability.

Several areas would benefit from greater clarity and refinement to strengthen the manuscript’s overall impact and generalizability. The scope and context of the work could be more explicitly framed at the outset. Because medical theses are not a universal requirement, it would be helpful for the introduction to clearly state upfront where and at what levels of medical education (e.g., undergraduate, graduate, residency) such theses are typically required – particularly in regions such as Europe, Asia, Africa, and Latin America. Relatedly, the authors should clarify how the proposed framework may be applicable, or adaptable, in settings where medical theses are not a standard requirement. Additional clarity and supporting context are also needed for the claim that master’s theses have low rates of conversion into publications.

Methodologically, the protocol is generally clear and well organized, but additional detail would improve transparency and reproducibility. For example, a brief explanation of the Delphi consensus process, including who participates, how meetings are facilitated, and how consensus is defined and managed if not reached, would strengthen the rigor of the approach. The rationale for providing participants with prior rankings should also be explained, along with any steps taken to mitigate potential bias across Delphi rounds. Similarly, the pilot study would benefit from clarification regarding the selection of the 20–25 students, whether participation to use the THESES-M guidelines was voluntary or mandatory, how consent was obtained, and how potential self-selection bias was considered. That is, were all eligible students required to use the THESES-M guidelines and were asked to volunteer to complete the survey?

Additional clarity in several areas is recommended:

1. The manuscript would benefit from clearer operational definitions, including concrete examples (e.g., what constitutes a “medical thesis”), and more specificity regarding purposive sampling (e.g., types of researchers and definitions of undergraduate versus postgraduate in this context).

2. The literature review process should specify the systematic procedures used (such as PRISMA), and the authors should clarify how documentation and data management will occur.

3. Additionally, the decision not to conduct a formal quality appraisal warrants justification.

4. Providing brief explanations of the statistical measures used, such as kappa and Kendall’s coefficients, would also improve accessibility for a broader audience.

5. The inclusion of a visual summary (e.g., a figure or flow diagram) of the protocol would help readers more readily grasp the overall process. Finally, while the framework is promising, it is currently limited to a single institutional context, and this should be acknowledged more explicitly as a limitation in terms of generalizability.

Overall, the study is feasible and addresses a meaningful gap in the field. With improved clarity in scope, enhanced methodological transparency, and some tightening to reduce repetition, the manuscript would be well positioned to make a valuable contribution to the standardization of medical thesis development and evaluation.

Reviewer #5: General Assessment The proposed protocol, THESES-M, aims to establish a reporting standard for medical theses, designed for inter-institutional application across various disciplines within the health sciences. This is a laudable goal that aligns perfectly with the rigorous, open-science mandate of PLOS ONE. Establishing these standards will be a highly beneficial undertaking for medical supervisors, researchers, and students globally, and their eventual publication in PLOS ONE will ensure the resulting guidelines are universally available.

Overall, the protocol is methodologically robust and closely adheres to the recognised guidance for guideline development set out by Moher et al. and the EQUATOR Network. It represents a strong candidate for publication. The following minor revisions and methodological clarifications are offered primarily for the benefit of the authors to further strengthen the study's reproducibility and psychometric transparency.

Major Comments

Protocol Timeline and PLOS ONE Compliance: It is understood from the text that data collection and the Delphi rounds have not yet commenced, which satisfies PLOS ONE’s prospective requirement for study protocols. However, to ensure immediate clarity for the editorial board and future readers regarding the prospective nature of the work, presenting the anticipated study timeline as a formal table or Gantt chart is highly recommended.

Considerations for the Likert Scale: The protocol proposes utilising a 10-point Likert-like scale to rate initial items, which is subsequently grouped into four operational categories. While there is precedent for wider scales in healthcare consensus methodologies (such as the 9-point RAND/UCLA appropriateness method), collapsing a 10-point ordinal scale into four distinct bins may introduce unnecessary variance and limit the granularity the scale was originally intended to capture. The authors might consider whether a more common 5- or 7-point scale would be more psychometrically stable for consensus thresholding. If the 10-point-to-4-bin structure is retained, a brief justification of its statistical validity and thresholding mechanism would strengthen the methodology.

Reproducibility of the Literature Review: To ensure the resulting guideline is truly applicable across diverse global institutions, the initial literature review phase should be explicitly defined in terms of its linguistic scope (e.g., clarifying whether Spanish or other non-English literature will be included). Furthermore, while a full formal scoping review may be out of scope for initial item generation, depositing the exact Boolean search strings, database limits, and search dates as a Supplementary File would greatly enhance the study's reproducibility.

Delphi Panel Attrition: Longitudinal Delphi panels frequently experience attrition across multiple rounds. While the protocol defines the intended initial panel size, it would benefit from a quantified a priori coping plan based on absolute numbers. To protect the statistical validity of the final consensus, the authors are encouraged to define their minimum acceptable absolute panel size (e.g., stating that consensus will not be considered valid if the panel drops below N = 20) rather than relying solely on optimal initial recruitment numbers.

Minor Comments

Data Availability: The authors state that de-identified data will be made available upon study completion, which is the correct approach for a study protocol prior to data collection. However, to ensure seamless compliance with PLOS ONE’s strict Data Availability policies, the authors should explicitly state where or how they intend to make this data available in the future (e.g., indicating that it will be deposited in a specific public repository such as OSF or Figshare, or published as Supplementary Information alongside the final manuscript).

Pilot Testing Phase: Please ensure that the pilot testing phase (Phase 4), which is mentioned in the abstract, is described with sufficient detail in the full text. Specifically, as this is likely a qualitative usability exercise, the protocol should note the target number of users anticipated to reach data saturation (e.g., 5-10 users) and detail the specific evaluation metrics (e.g., clarity, feasibility) that will be applied to the consensus-based checklist and the explanation and elaboration document.

**********

7. PLOS authors have the option to publish the peer review history of their article (what does this mean?). If published, this will include your full peer review and any attached files.

If you choose “no”, your identity will remain anonymous but your review may still be made public.

**Do you want your identity to be public for this peer review?**  For information about this choice, including consent withdrawal, please see our Privacy Policy.

Reviewer #1: No

Reviewer #2: No

Reviewer #3: **Yes:**  SAKINAH AWANGHARUN

Reviewer #4: No

Reviewer #5: **Yes:**  Francesco Bolstad

**********

[NOTE: If reviewer comments were submitted as an attachment file, they will be attached to this email and accessible via the submission site. Please log into your account, locate the manuscript record, and check for the action link "View Attachments". If this link does not appear, there are no attachment files.]

To ensure your figures meet our technical requirements, please review our figure guidelines: https://journals.plos.org/plosone/s/figures

You may also use PLOS’s free figure tool, NAAS, to help you prepare publication quality figures: https://journals.plos.org/plosone/s/figures#loc-tools-for-figure-preparation.

NAAS will assess whether your figures meet our technical requirements by comparing each figure against our figure specifications.

Attachment Submitted filename: THESES-M_ protocol.pdf

Attachment Submitted filename: Reviewer comments PLOS.docx

# Author comment: Author response to Decision Letter 1

**DOI:** 10.1371/journal.pone.0354899.r002

20 Jun 2026

Dear Editor,

We have revised the manuscript according to the reviewers’ comments. A detailed point-by-point response to all reviewer comments has been uploaded as a separate file titled “Response to reviewers PLOS ONE R1.” The revised manuscript, clean version, marked-up version, cover letter, and updated figures have also been uploaded.

Sincerely,

Geovani López-Ortiz

Attachment Submitted filename: Response to reviewers PLOS ONE R1.pdf

# Editor report: Decision Letter 1

**DOI:** 10.1371/journal.pone.0354899.r003

**Authors:** Paolo Landa

15 Jul 2026

THESES-M: protocol for the development of a reporting guideline for medical theses

PONE-D-26-15953R1

Dear Dr. López-Ortiz,

We’re pleased to inform you that your manuscript has been judged scientifically suitable for publication and will be formally accepted for publication once it meets all outstanding technical requirements.

Within one week, you’ll receive an e-mail detailing the required amendments. When these have been addressed, you’ll receive a formal acceptance letter and your manuscript will be scheduled for publication.

An invoice will be generated when your article is formally accepted. Please note, if your institution has a publishing partnership with PLOS and your article meets the relevant criteria, all or part of your publication costs will be covered. Please make sure your user information is up-to-date by logging into Editorial Manager at Editorial Manager® and clicking the ‘Update My Information' link at the top of the page. For questions related to billing, please contact billing support.

If your institution or institutions have a press office, please notify them about your upcoming paper to help maximize its impact. If they’ll be preparing press materials, please inform our press team as soon as possible -- no later than 48 hours after receiving the formal acceptance. Your manuscript will remain under strict press embargo until 2 pm Eastern Time on the date of publication. For more information, please contact onepress@plos.org.

Kind regards,

Paolo Landa, Ph.D.

Academic Editor

PLOS One

Additional Editor Comments (optional):

the authors have well addressed all the questions raised by the reviewers.

Reviewers' comments:

# Editor report: Acceptance letter

**DOI:** 10.1371/journal.pone.0354899.r004

**Authors:** Paolo Landa

PONE-D-26-15953R1

PLOS One

Dear Dr. López-Ortiz,

I'm pleased to inform you that your manuscript has been deemed suitable for publication in PLOS One. Congratulations! Your manuscript is now being handed over to our production team.

At this stage, our production department will prepare your paper for publication. This includes ensuring the following:

* All references, tables, and figures are properly cited

* All relevant supporting information is included in the manuscript submission,

* There are no issues that prevent the paper from being properly typeset

You will receive further instructions from the production team, including instructions on how to review your proof when it is ready. Please keep in mind that we are working through a large volume of accepted articles, so please give us a few days to review your paper and let you know the next and final steps.

Lastly, if your institution or institutions have a press office, please let them know about your upcoming paper now to help maximize its impact. If they'll be preparing press materials, please inform our press team within the next 48 hours. Your manuscript will remain under strict press embargo until 2 pm Eastern Time on the date of publication. For more information, please contact onepress@plos.org.

You will receive an invoice from PLOS for your publication fee after your manuscript has reached the completed accept phase. If you receive an email requesting payment before acceptance or for any other service, this may be a phishing scheme. Learn how to identify phishing emails and protect your accounts at https://explore.plos.org/phishing.

If we can help with anything else, please email us at customercare@plos.org.

Thank you for submitting your work to PLOS ONE and supporting open access.

Kind regards,

PLOS ONE Editorial Office Staff

on behalf of

Dr. Paolo Landa

Academic Editor

PLOS One
